# Volumetric MRI and FDG-PET hypometabolism biomarkers of frontotemporal dementia: protocol for a systematic review and meta-analysis

**DOI:** 10.1136/bmjopen-2025-101729

**Published:** 2025-12-12

**Authors:** Taylor J Solomon, Ana Antonic-Baker, Lorena Romero, Benjamin Sinclair, Terence J O’Brien, Lucy Vivash

**Affiliations:** 1 Department of Neuroscience, Monash University, Melbourne, Victoria, Australia; 2 Ian Potter Library, Alfred Health, Melbourne, Victoria, Australia; 3 Department of Neurology, Alfred Health, Melbourne, Victoria, Australia; 4 Department of Neurology, Royal Melbourne Hospital, Melbourne, Victoria, Australia; 5 Department of Medicine, The University of Melbourne, Melbourne, Victoria, Australia

**Keywords:** Dementia, Magnetic resonance imaging, Neuropathology, Diagnostic radiology

## Abstract

**Abstract:**

**Introduction:**

Frontotemporal dementia (FTD) remains challenging to diagnose owing to the marked clinical heterogeneity associated with the disease. This heterogeneity stems from the complex interplay of various clinical phenotypes, genetic mutations and underlying neuropathologies, such as TDP-43 and tau proteinopathies. Currently, there is no single confirmed biomarker that can reliably diagnose disease, specifically disease stage, disease subtype and underlying neuropathology. Recent research has indicated that neuroimaging techniques hold the most promise for the discovery of FTD biomarkers. We propose a protocol for a systematic review and meta-analysis to identify MRI and fluorodeoxyglucose positron emission tomography (FDG-PET) biomarkers associated with clinical, genetic and pathological subtypes of FTD. We aim to address the following research questions: can regional MRI volumetry and FDG-PET hypometabolism differentiate (1) FTD patients from healthy controls; (2) sporadic cases of FTD from healthy controls; (3) genetic cases of FTD (MAPT, GRN, and C9orf72 mutations); and (4) underlying neuropathology, specifically discriminating between tau- and TDP-43-based FTD?

**Methods:**

Literature searches will be performed across three databases: Ovid Medline, Ovid Embase and Web of Science. Publications that have fewer than five participants, are non-human-based, not written in the English language or contain unpublished data will be excluded. Two independent investigators will screen and subsequently evaluate which publications to include. Should any disagreements arise, a third investigator will settle the discrepancy. After the random-effects meta-analysis has been used to extract and pool the data, I^2^ analysis will be used to quantify heterogeneity.

**Ethics and dissemination:**

Ethics approval will not be required for this research. On completion, the systematic review and meta-analysis will be published in a peer-reviewed journal.

**PROSPERO registration number:**

CRD42024545302.

**STRENGTHS AND LIMITATIONS OF THIS STUDY**

- Inclusion of genetically and pathologically confirmed cases of frontotemporal dementia (FTD) - the inclusion of genetic and/or pathologically confirmed cases of FTD will enhance the robustness and reliability of volumetric MRI and fluorodeoxyglucose positron emission tomography (FDG-PET) hypometabolism biomarker associations with FTD subtypes.
- Utilisation of both structural and functional imaging modalities - this study incorporates volumetric MRI and FDG-PET hypometabolism to evaluate the complementary utility of structural and functional imaging in delineating FTD subtypes, thereby offering a more comprehensive assessment of biomarker potential.
- Potential variability in FTD clinical and pathological subtype reporting - FTD encompasses a heterogeneous spectrum of clinical syndromes and genetic/neuropathological subtypes, some of which may not be consistently reported across the included studies, potentially impacting the accuracy of subgroup analyses.
- Evolving diagnostic criteria - clinical understanding and subtype diagnosis of FTD has improved over time; therefore, earlier research may have used less accurate and/or outdated diagnostic criteria, potentially resulting in subtype misclassification.
- Publication and/or reporting bias and lack of individual-level data - studies included for analysis may selectively report significant findings, and the absence of individual-level data limits the ability to independently verify results and harmonise outcomes across studies.

## Introduction

 Frontotemporal lobar degeneration (FTLD) is an overarching neuropathological term which describes a spectrum of diseases marked by neurodegeneration of the frontal and temporal lobes of the brain.^1^ Frontotemporal dementia (FTD) is a cluster of clinical syndromes that are characterised by this neuropathology, resulting in symptoms such as behavioural abnormalities, diminished executive control and social-cognitive function, language deficits, and motor impairments.^2^

The spectrum of FTD syndromes is associated with a diverse range of neuropathological processes, including proteinopathies predominantly involving tau and TDP-43 accumulation.^3 4^ A modest number of FTD patients present with rarer proteinopathies, including ubiquitin accumulations, as well as fused-in-sarcoma (FUS) pathologies.^5 6^ Disease progression is further influenced by cellular processes such as neuroinflammation.^7^ Importantly, the relationship between FTD clinical syndrome and underlying neuropathology is highly complex, as different neuropathologies often contribute to multiple FTD syndromes and clinical phenotypes.^8^

FTD can present as either sporadic or genetic, with approximately 30% of cases linked to an autosomal dominant mutation.^9^ Familial FTD is commonly attributed to mutations in one of three genes: MAPT, GRN and C9orf72. Among these mutations, genotype-neuropathology correlations indicate that MAPT mutations are linked with tau pathology, while C9orf72 and GRN mutations are associated with TDP-43 proteinopathies.^10^,^12^ Several rare genetic mutations observed in FTD include TANK-binding kinase 1 (TBK1), charged multivesicular body protein 2B (CHMP2B), valosin-containing protein (VCP) and FUS.^13 14^

Although FTD arises from distinct neuropathologies and genetic mutations, there remains substantial clinical and pathological overlap across the spectrum.^15^ The interplay between proteinopathies, genetic mutations, and cellular processes contributes to the extreme complexity and heterogeneity that define FTD as hallmarks of disease.^16^ Specifically, the unclear relationship between clinical phenotype and underlying neuropathology results in blurred boundaries between syndromes, posing significant challenges for the timely and accurate discrimination and diagnosis of specific FTD subtypes.^17 18^ At present, there is no single confirmed biomarker that can reliably diagnose clinical syndrome and neuropathology with high specificity and sensitivity in vivo.^19^ The ability to diagnose underlying proteinopathies is particularly crucial for advancing future therapeutics, as it will facilitate appropriate treatment strategies that are targeted at specific neuropathological presentations rather than clinical FTD syndromes which may be associated with multiple independent pathologies.^20^

Neuroimaging holds significant promise in identifying FTD biomarkers. MRI volumetry is arguably one of the most widely used and informative neuroimaging techniques in FTD diagnosis, offering vital insights into brain atrophy patterns that can differentiate FTD from other neurodegenerative diseases.^21^ Frontal and temporal lobe atrophy on MRI is an established sign of FTLD, but has limited utility in differentiating different clinical syndromes or underlying pathology.^22^ Nonetheless, differences in patterns of atrophy may be able to differentiate distinct FTD subtypes; for example, the behavioural variant (bvFTD) and primary progressive aphasia (PPA).^23^ Atrophy in bvFTD primarily impacts the frontal lobe and subcortical regions, resulting in behavioural abnormalities and executive dysfunction.^24^ By contrast, atrophy observed in PPA demonstrates a more localised pattern which is primarily restricted to specific regions of the temporal and parietal lobes, thus corresponding with language-specific impairments seen in this subtype.^23^

Fluorodeoxyglucose positron emission tomography (FDG-PET) is a complementary neuroimaging modality that may be additionally implemented on clinical suspicion of FTD.^25^ Previous research has revealed characteristic hypometabolism patterns that can effectively discriminate between FTD syndromes such as bvFTD and PPA, with patterns of hypometabolism closely mirroring that of structural atrophy.^25 26^ Notably, hypometabolism patterns may reveal additional functional loss in brain regions that appear structurally preserved on MRI.^27^ Hence, FDG-PET offers a valuable tool for the early detection and diagnosis of FTD.^28^ Furthermore, similarly to MRI volumetry, although FDG-PET hypometabolism may differentiate specific FTD syndromes, its utility in reliably delineating between underlying neuropathologies has yet to be established and warrants further investigation.^28^

Combining structural and functional imaging allows for a more comprehensive clinical assessment of the condition. Indeed, it has been postulated that combining MRI volumetry and FDG-PET improves FTD subtype discrimination and diagnosis.^29^ Thus, these neuroimaging modalities offer a timely, non-invasive, readily accessible means of detecting and predicting FTD-specific brain changes indicative of underlying neuropathology.^29^ The ability to predict TDP-43 and tau pathology using neuroimaging is crucial, as it is likely these proteinopathies require therapeutics with distinct mechanisms of action, thus allowing for precision medicine approaches such as pathology-based treatment. The capacity to classify patients according to their predominant proteinopathies will additionally facilitate accurate patient stratification and enrolment into clinical trials evaluating novel FTD therapeutics. Therefore, while existing data support the use of MRI volumetry and FDG-PET hypometabolism as potential biomarkers for preliminary FTD diagnosis, in order to fully incorporate these diagnostic tools into clinical practice, additional research is required to validate their discriminatory power between clinical syndrome and neuropathological substrates.

The proposed systematic review and meta-analysis will address the following research questions:

1. Can regional MRI volumetry and FDG-PET hypometabolism differentiate FTD patients from healthy controls, thereby supporting early and accurate FTD diagnosis upon clinical presentation?
2. Can regional MRI volumetry and FDG-PET hypometabolism differentiate sporadic cases of FTD from healthy controls, improving diagnostic accuracy in the absence of known genetic influence - which accounts for the large majority of cases - and enhancing our understanding of the disease mechanisms underscoring sporadic FTD?
3. Can regional MRI volumetry and FDG-PET hypometabolism differentiate genetic cases of FTD, specifically delineating the three primary genotypes - MAPT, GRN, and C9orf72 - most commonly associated with FTD, thus informing genotype-specific disease trajectories and prognosis?
4. Can regional MRI volumetry and FDG-PET hypometabolism differentiate underlying neuropathology, specifically discriminating between tau- and TDP-43-based FTD, hence facilitating appropriate patient stratification in clinical trials and pathology-targeted therapeutic intervention?

## Methods and analysis

### Population

The population used in this study will be adults with a clinical diagnosis of FTD, including all syndromes under the FTLD umbrella - for example, PPAs, bvFTD, progressive supranuclear palsy, corticobasal syndrome and amyotrophic lateral sclerosis. All studies including participants with a diagnosis will be included, irrespective of the diagnostic criteria used or the certainty of the diagnosis. Participants with genetic mutations associated with FTDs but who are asymptomatic will be excluded from the analyses.

### Intervention

The intervention to be studied will be reduced brain volume as measured by MRI, demonstrated as a reduction compared with a comparator group (controls and patient subgroups). Similarly, hypometabolism as measured by FDG-PET will be studied, as demonstrated by a reduction in metabolism compared with a comparator group (controls and patient subgroups). Visual, region of interest (ROI), and global analyses - including voxel-based morphometry (VBM) and statistical parametric mapping (SPM) - will be included.

### Control population

Cases of FTD will be compared with healthy controls. Sub-analyses will be performed based on individual clinical diagnoses, pathology and presence of genetic mutations. Analyses will compare each patient (sub-)group to a control group, as well as comparisons between patient groups with differing characteristics; for example, clinical syndrome and proteinopathy.

### Outcome

The main outcomes investigated will be the ability and accuracy of MRI volumetry and FDG-PET hypometabolism biomarkers in identifying FTD in comparison to healthy controls. We will focus our analysis particularly on the ability of these imaging modalities to detect patterns of cortical atrophy and hypometabolism in ROIs, such as the frontal and temporal lobes. Furthermore, we will compare FTD with distinct neuropathologies, including TDP-43 and tau proteinopathies, as well as genetic mutations, including MAPT, GRN, and C9orf72. Thus, we will evaluate whether specific patterns of cortical atrophy and hypometabolism can accurately delineate FTD clinical and pathological subtypes.

### Search strategy

Following the development and piloting of the search strategy, the final searches will be conducted by an experienced research librarian on three databases: Ovid Medline (Medical Literature Analysis and Retrieval System Online), Ovid Embase (Excerpta Medica Database) and Web of Science (Clarivate). The search strategy will use a combination of database-specific subject headings and free text terms that will cover three concept areas: (1) Concept A - FTD/FTLD; (2) Concept B - volumetric/structural MRI or Fluorodeoxyglucose positron emission tomography; and (3) Concept C - brain volume changes/hypometabolism/neuropathologies. Representative search terms for each concept are summarised in table 1, while the full search strategies are provided in online supplemental file. Forward and backward citation tracking will also be undertaken. There are no restrictions on the date of publication; however, studies will be limited to the English language. In order to ensure the search is comprehensive and exhaustive, the search will be updated before review completion.

**Table 1:** Summary of search concepts and representative terms for volumetric MRI and FDG-PET hypometabolism biomarkers in FTD

Concept	Representative terms/Boolean operators
Frontotemporal dementia and related syndromes	“frontotemporal dementia” OR FTD OR “behavioural variant FTD” OR bvFTD OR “Pick’s disease” OR “primary progressive aphasia” OR PPA OR “semantic variant PPA” OR svPPA OR “non-fluent variant PPA” OR nfvPPA OR “logopenic variant PPA” OR lvPPA OR “progressive non-fluent aphasia” OR PNFA OR “semantic dementia” OR SD OR “corticobasal syndrome” OR CBS OR “progressive supranuclear palsy” OR PSP OR “motor neuron disease” OR ALS OR “FTD-MND” OR “ALS-MND”
Volumetric/structural MRI	MRI OR “volumetric MRI” OR “structural MRI” OR “T1-weighted MRI” OR T1WI OR “brain volumetry” OR Structural MRI OR functional MRI
FDG-PET	FDG OR [18F]FDG OR “FDG-PET” OR “F-18 FDG PET” OR “radiotracer PET” OR Fluorodeoxyglucose
Brain changes (structural and functional)	“brain atrophy” OR “regional atrophy” OR “temporal lobe atrophy” OR “frontal lobe atrophy” OR “hippocampal volume” OR “parietal volume” OR “cerebellar volume” OR “subcortical volume” OR “gray matter loss” OR “white matter loss” OR “cortical volume” OR “subcortical volume” OR “functional/metabolic changes” OR “hypometabolism” OR “hypermetabolism” OR “glucose metabolism alterations” OR “brain metabolism patterns”

ALS, amyotrophic lateral sclerosis; bvFTD, behavioural variant frontotemporal dementia; CBS, corticobasal syndrome; FDG, fluorodeoxyglucose; FTD, frontotemporal dementia; lvPPA, logopenic variant PPA; MND, motor neuron disease; nfvPPA, non-fluent variant PPA; PET, positron emission tomography; PNFA, progressive non-fluent aphasia; PPA, primary progressive aphasia; PSP, progressive supranuclear palsy; SD, semantic dementia; svPPA, semantic variant PPA; T1WI, T1-weighted imaging.

### Selection process

Retrieved studies will be managed using Covidence software. Following the removal of duplicates, all studies will be screened using the title and abstract by two independent investigators. Inclusion criteria will be: (1) publications which describe randomised control trials, cohort studies and case–control studies which may be prospective or retrospective; (2) publications with greater than or equal to five participants; (3) publications which describe participants having undergone MRI and/or FDG-PET; and (4) structural volumes (MRI) and/or measures of metabolism (FDG-PET) have been reported. Exclusion criteria will include: (1) publications in languages other than English; (2) non-human studies; (3) publications which report on unpublished data; (4) publications with fewer than five participants; and (5) publications that include only VBM or SPM without numeric results. Any disagreements regarding eligibility for full-text inclusion will be resolved by a third investigator. The full-text reviews will be undertaken independently by two investigators, and any disagreements regarding eligibility for inclusion in the analysis will be resolved by a third investigator. Data will be extracted by two independent investigators into a pre-defined data extraction spreadsheet. We will contact the authors for further information if the relevant data is not reported in the original publication. The data extraction results will be compared after the first ten publications, and if congruent, the remainder of the data collection will be performed by a single investigator. Discrepancies will be resolved by discussion and/or by a third investigator.

### Data collection

For this research, a wide range of patient- and study-specific information will be collected. This will contain publication specifics, including the research journal in which it was published, the first author’s name, and the year of publication. The demographics of the patients, including age, gender, and any other pertinent information that may affect the study’s results, will be documented. The specific FTD variant (bvFTD, PPA etc) and, where available, the diagnostic criteria used, related genetic mutations (MAPT, GRN, C9orf72, or others), and the degree of diagnostic certainty (for example, “definite”, “probable”, and “possible”) will be examined. Where available, the underlying disease pathology confirmed via autopsy or genetic testing will be extracted, including detailed information about the FTD diagnosis. The clinical presentation of the patients, such as the onset, nature, severity of symptoms (measured by appropriate clinical scales), as well as the years from symptom onset to diagnosis, will additionally be recorded.

Furthermore, comprehensive details on the MRI and FDG-PET scans will be collated. This will contain information on the precise imaging procedures used for MRI, including MRI sequences employed, the magnet strength, the scan resolution, the regions of the brain that are examined, as well as the techniques used for analysis (visual review, ROI volumetry, and VBM). Regarding FDG-PET scan procedures, we will record the scanner type, the scan resolution, the regions of the brain that are examined, as well as the techniques used for analysis (visual review, ROI analysis, and SPM). In instances of missing data, analysis will proceed using the available data only, with contributing study and participant counts explicitly specified for each analysis.

### Risk of bias assessment

Cochrane Collaboration’s Risk of Bias 2.0 (RoB 2) tool will be used for randomised studies. The RoB 2 proposes a framework for evaluating the risk of bias for a randomised study.^30^ It measures bias across five central domains: bias arising from the randomisation process, bias due to deviations from intended interventions, bias due to missing outcome data, bias in the measurement of the outcome, and bias in the selection of the reported result. For each domain, the framework poses specific signalling questions, and each domain is subsequently assessed as low risk, moderate risk or high risk of bias. Combining the evaluations from each domain yields the study’s total risk of bias. Dependent on the perceived severity of the risks, studies recorded as high risk across one or more domains may indicate a high overall risk of bias, whereas studies recorded as low risk across several domains are considered to have a low overall risk of bias. This ensures a transparent and comprehensive assessment of the quality of randomised controlled trials.

The Newcastle Ottawa Scale (NOS) will be used for non-randomised studies. The NOS is a tool used for appraising the quality of non-randomised research, specifically cohort and case–control studies.^31^ It is used to assess bias risk across three primary domains: Selection, Comparability, and Outcome (or Exposure). Based on categories, each study is assigned a star rating. The Selection domain evaluates the ability of a study to define and select participants, awarding up to four stars. The Comparability domain grades how effectively the research controls for possible sources of confounding variables, awarding up to two stars. For cohort studies, the Outcome domain (or Exposure for case–control studies) examines the methodology used to assess and report study outcomes, awarding up to three stars. The ratings across the three domains are totalled, and based on the total score, studies are rated as high (seven to nine stars), moderate (five to six stars) or low quality (less than five stars). Thus, a maximum of nine stars may be awarded to a study, whereby a greater star rating indicates higher quality and decreased risk of bias. This process will be repeated for all included studies whereby two reviewers in consultation will determine final scores for the total bias outcome.

### Data analysis

For each study, we will calculate the effect size, which is the population that is correctly identified as having FTD according to imaging measures. We will then use DerSimonian and Laird random-effects meta-analysis to calculate the overall effect size of the favourable outcome, including its 95% confidence intervals. The heterogeneity will be calculated with Cochran’s Q test, which evaluates whether the observed differences are attributed to chance, and I^2^ statistics, which estimate the proportion of variability caused by heterogeneity as opposed to random variation.^32 33^ All analyses will be conducted using Stata, and an I^2^ value greater than 75% will be indicative of substantial heterogeneity. To explore the sources of heterogeneity, we will perform a meta-regression on the subset of data including FTD clinical syndrome (bvFTD, PPAs, etc.), genetic mutations (MAPT, GRN, C9orf72, or others), confirmed underlying neuropathology (tau or TDP-43), and imaging modality (MRI and/or FDG-PET). To assess small-study effects and publication bias, we will use Egger regression, funnel plots, and trim-and-fill analysis.^34^

Studies will be incorporated irrespective of the diagnostic criteria used and diagnostic certainty categorisation in order to ensure comprehensive coverage of the literature. However, to account for differing levels of diagnostic certainty across studies, we intend to stratify analyses to separately examine FTD cases with a “definite” and “probable” diagnoses, as well as stratifying according to the diagnostic criteria used. This will enable assessment of whether diagnostic criteria and certainty influence biomarker discovery.

Analyses will be conducted within imaging modalities, with ROI and VBM findings analysed independently to account for methodological differences. To ensure comparability across included studies, analyses will examine ROIs as reported by each study. Thus, we aim to align findings based on equivalent anatomical regions wherever possible as opposed to attempting transformations across differing brain atlases or registration conventions. SPM and VBM findings will be reported following the standard convention, using either coordinates or anatomical labels. Regional summaries will also be harmonised by mapping results to the most commonly used brain atlas in the dataset. Moreover, qualitative analysis will summarise findings across modalities and analysis methods.

There will be a minimum threshold of five papers per subgroup for inclusion in quantitative analyses; results from subgroups with fewer papers than this will instead be qualitatively synthesised in order to avoid unstable statistical estimates. Finally, the risk of bias scores will be incorporated into sensitivity analyses to assess the robustness of the review’s findings. Studies with a higher risk of bias will be excluded or analysed separately to determine whether their inclusion significantly influences the overall results and conclusions.

## Patient and public involvement

Neither individuals with lived experience of FTD, nor patient advocacy groups or other community groups were involved in the design, conduct, reporting, or dissemination plans of this research. However, following publication, dissemination to these audiences may be undertaken to ensure accessibility of research and broader community engagement, such as presentation of research findings at relevant scientific conferences and other clinical and academic forums. Study findings may also be presented at public-facing events through our institutes and patient advocacy and support groups, such as Dementia Australia and Fight Parkinson’s Victoria.

## Ethics and dissemination

The proposed protocol for a systematic review and meta-analysis does not require ethics approval, as the data to be collected cannot be connected to individual patients. It is intended that this systematic review and meta-analysis will be published in a high-quality, high-impact peer-reviewed journal.

## Discussion

The aim of this systematic review and meta-analysis is to investigate volumetric MRI and FDG-PET hypometabolism biomarkers that can be used to differentiate between FTD clinical syndromes, as well as between FTD and healthy controls. The study additionally aims to investigate the ability of these imaging biomarkers to specifically distinguish between different genetic mutations and underlying neuropathologies. In doing so, this research will assess the clinical utility of MRI volumetry and FDG-PET as imaging modalities in the accurate diagnosis of FTD syndromes. To our knowledge, this will be the first systematic review and meta-analysis investigating volumetric MRI and FDG-PET hypometabolism biomarkers specifically in genetically or pathologically confirmed cases of FTD. Notably, prior meta-analyses have not reliably delineated between pathologically or genetically confirmed and clinically diagnosed cases, a distinction we consider essential for accurately evaluating the robustness of biomarkers. Nonetheless, in an effort to offer vital insights into the structural and metabolic alterations observed in the disease, this study strives to expand on earlier findings that have emphasised the merit of neuroimaging techniques in FTD research.

The clinical relevance of this research lies in its potential to improve diagnostic and prognostic approaches for FTLD. This study seeks to offer strong evidence that can guide clinical decision-making by collating valuable data on volumetric MRI and FDG-PET hypometabolism biomarkers for cases across the FTLD spectrum. Such research may aid in the development of refined diagnostic criteria that encompass identified biomarkers to increase diagnostic certainty between FTD subtypes, which in turn will support new therapeutic approaches and provide measures of disease progression. For instance, conducting clinical trials for novel FTD therapeutics presents substantial challenges. The extensive clinical and pathological overlap associated with FTD leads to poor patient stratification and limits the ability to efficiently assess treatment effects.^35^ Currently, many clinical trials investigating the efficacy of disease-modifying novel therapeutics targeting tau or TDP-43 recruit individuals with known genetic mutations to increase pathological certainty within cohorts. Examples include several monoclonal antibody trials, with AL001 tested in GRN-positive cohorts,^36^ and PBFT-02 tested in GRN- and C9orf72-positive cohorts.^37^ Other ongoing studies include tau-targeting therapies such as UCB0107^38^ and sodium selenate.^39^ To date, these studies have focused on FTD associated with TDP-43-related genetic mutations,^38^ as well as primary tauopathies such as progressive supranuclear palsy,^39^ respectively. Although this approach increases cohort homogeneity, it excludes a significant portion of sporadic FTD patients, reducing the generalisability of study findings and slowing advancements in FTD treatments.

Therefore, the outcomes of this research have direct implications for clinical trial design. The discovery of in vivo neuroimaging biomarkers that differentiate between FTD proteinopathies will facilitate pathology-based trial enrolment, rather than reliance on genetic status. Consequently, such biomarkers would improve stratification of sporadic FTD cases by likely underlying pathology, enabling the evaluation of pathology-targeted therapies in individuals whose pathology cannot be inferred with confidence. Furthermore, these biomarkers may enhance endpoint selection, monitoring of disease progression, and accurate detection of drug effects. FTD clinical trials may therefore move towards a precision medicine paradigm that mirrors developments observed in other neurodegenerative diseases.

This study carries inherent limitations. FTLD encompasses a suite of clinical syndromes, as well as pathological and genetic subgroups, some of which may not be consistently reported or distinguished in the included research. Similarly, understanding and subsequent diagnosis of FTD have improved over time.^40^ Less nuanced methods may have been employed in earlier research, possibly leading to inaccurate categorisation or incomplete clinical descriptions of FTD cases, as well as diagnostic delays. As such, disease stage at the time of imaging may differ across studies, potentially influencing volumetric and metabolic measurements. Finally, publication and reporting bias may be present, and the lack of individual-level data restricts the ability to validate reporting consistency between studies. Ultimately, this research aims to consolidate and extend existing evidence on MRI and FDG-PET biomarkers in FTD, translating these findings into clinically meaningful tools for pathology-driven diagnosis, prognosis, and therapeutic intervention.

## Supplementary material

10.1136/bmjopen-2025-101729 online supplemental file 1
