# Supplementary material for: Volumetric MRI and FDG-PET hypometabolism biomarkers of frontotemporal dementia: protocol for a systematic review and meta-analysis
Source: BMJ Open. 2025 Dec 12;15(12):e101729. doi: 10.1136/bmjopen-2025-101729 (PMC12706253; doi:10.1136/bmjopen-2025-101729)
Supplement: online supplemental file 1 [file bmjopen-15-12-s001.docx]

**Volumetric MRI and FDG-PET hypometabolism biomarkers of Frontotemporal Dementia: Protocol for a systematic review and meta-analysis.**

**Supplementary data**

Literature Search Strategy

**DATABASE 1: MEDLINE**

Ovid MEDLINE(R) <1946 to August 27, 2024>

| **#** | **Query** |
| --- | --- |
| 1 | aphasia, primary progressive/ or frontotemporal lobar degeneration/ or frontotemporal dementia/ or "pick disease of the brain"/ or primary progressive nonfluent aphasia/ or corticobasal degeneration/ or supranuclear palsy, progressive/ or motor neuron disease/ or amyotrophic lateral sclerosis/ |
| 2 | RNA-Binding Protein FUS/ or C9orf72 Protein/ or TDP-43 Proteinopathies/ or tau Proteins/ or Tauopathies/ |
| 3 | (frontotemporal lobar degeneration or fronto temporal lobar degeneration or frontal dementia or frontal lobe dementia or Pick* complex or frontotemporal dementia or frontal-temporal dementia or fronto temporal dementia or behavio?ral variant FTD or frontal variant FTD or Pick* disease or Pick dementia or Pick syndrome or FTD-behavio?ral variant* or FTD-language variant* or amyotrophic lateral scleros?s or ALS dementia or motor neuron disease* or sporadic MND or motoneuron disease* or motor neurone disease* or motor system disease* or primary lateral scleros?s or corticobasal syndrome* or corticobasal degeneration or cortical basal degeneration or cortical basal ganglionic degeneration or corticobasal ganglionic degeneration or cortico basal ganglionic degeneration or primary progressive aphasia* or temporal variant FTD or PPA syndrome* or semantic aphasia* or logopenic variant PPA or logopenic progressive aphasia* or "progressive non-fluent aphasia*" or "Non-fluent progressive aphasia*" or nonfluent progressive aphasia* or progressive nonfluent aphasia* or "non-fluent primary progressive aphasia" or semantic dementia or "Non-fluent?agrammatic variant*" or Nonfluent?agrammatic variant* or semantic PPA or Semantic variant PPA or progressive supranuclear palsy or Steele-Olszewski-Richardson Syndrome or steele richardson olszewski syndrome or supranuclear progressive palsy or PSP-Richardson* syndrome or "PSP-pure akinesia with gait freezing" or PSP-Progressive gait freezing or "PSP-Speech and Language variant" or PSP-Speech?Language or PSP-Frontal or Frontal PSP or Progressive supranuclear ophthalmoplegia* or vertical supranuclear ophthalmoplegia* or supranuclear vertical gaze palsy or vertical supranuclear gaze palsy).mp. |
| 4 | (C9orf72 or MAPT or TDP43 or "TDP-43" or TARDBP43 or "TARDBP-43" or TAU protein or "fused in sarcoma" or FUS gene* or FUS protein or FUS RNA binding protein or "protein FUS" or "TAR DNA binding protein 43" or "C9 open reading frame 72" or "chromosome 9 open reading frame 72" or Tauopath* or "transactive response DNA binding protein 43").mp. |
| 5 | (bvFTD or fvFTD or tvFTD or svPPA or nfvPPA or lpPPA or lvPPA or PPA-L or PNFA or SemD or PPA-G or nfPPA or PPA-S or PSP-RS or PSPS or PSP-P or PAGF or PSP-PGF or PSP-SL or PSP-F or PSP-bvFTD or PSP-PNFA or PSP-C or vPSP or FTD-MND or ALS-MND or ALS-FTD or ALS?FTD or CBS-CBD or CBS-PSP or PSP-CBS or PSP-PAGF or FTLD-TDP or FTLD-tau or sFTLD or gFTLD or sMND or C9FTLD).mp. |
| 6 | 1 or 2 or 3 or 4 or 5 |
| 7 | Magnetic Resonance Imaging/ or Diffusion Magnetic Resonance Imaging/ or Diffusion Tensor Imaging/ |
| 8 | ((volumetric* or volumetry or volumes or structural change* or structural* alterat* or "change* in structure" or microstructural degradation or macrostructural atrophy) and (brain* or cerebral or temporal or cortical or subcortical or hippocamp* or parahippocamp* or frontal or parietal or temporoparietal or frontoparietal or cerebell* or intracranial or gr#y matter or white matter or ventricular or orbitofrontal or bifrontal or cortices or diencephalon or pallidum or amygdala* or frontotemporal or thalamus or subthalamus or neuroanatomical or third ventricle* or midbrain or parenchymal or thalami? or subthalami? or basal ganglia or prefrontal or temporopolar or angular gyrus or postcentral gyrus or precuneus or cingulate gyrus or gyrus rectus or anterior cingulate or posterior cingulate or corpus callosum or insula or claustrum or caudate or frontal gyrus or precentral gyrus or supplementary motor area or paracentral lobule or postcentral gyrus or pons or medulla oblongata or angular gyrus or supramarginal gyrus or posterior cingulum or motor cortex or fusiform gyrus or occipital or frontal cortex or nucleus accumbens or globus pallidus or putamen or primary cortex)).mp. |
| 9 | 6 and 7 and 8 |
| 10 | (((MRI? or Magnetic Resonance Imag* or MR imag* or MR scan* or Structural MRI or functional MRI or NMR imag* or T1 weighted imag* or T1-weighted MRI? or tensor-based morphometry or T1WI MRI or fMRI or FMRIB or sMRI or MRI assessment* or MRI stud* or MRI analys* or MRI-based or MRI measure* or MRI marker* or vMRI or Magnetic Resonance Imag* or morphometric MRI or MR? Biomarker*) adj7 (volumetric* or volumetry or volumes or structural change* or structural* alterat* or "change* in structure" or microstructural degradation or macrostructural atrophy)) and (brain* or cerebral or temporal or cortical or subcortical or hippocamp* or parahippocamp* or frontal or parietal or temporoparietal or frontoparietal or cerebell* or intracranial or gr#y matter or white matter or ventricular or orbitofrontal or bifrontal or cortices or diencephalon or pallidum or amygdala* or frontotemporal or thalamus or subthalamus or neuroanatomical or third ventricle* or midbrain or parenchymal or thalami? or subthalami? or basal ganglia or prefrontal or temporopolar or angular gyrus or postcentral gyrus or precuneus or cingulate gyrus or gyrus rectus or anterior cingulate or posterior cingulate or corpus callosum or insula or claustrum or caudate or frontal gyrus or precentral gyrus or supplementary motor area or paracentral lobule or postcentral gyrus or pons or medulla oblongata or angular gyrus or supramarginal gyrus or posterior cingulum or motor cortex or fusiform gyrus or occipital or frontal cortex or nucleus accumbens or globus pallidus or putamen or primary cortex)).mp. |
| 11 | 6 and 10 |
| 12 | ((MR scan* or MR imag* or Magnetic resonance imag* or Structural MRI or functional MRI or NMR imag* or T1 weighted imag* or T1-weighted MRI? or tensor-based morphometry or morphometric MRI or MR? Biomarker* or MRI measure* or fMRI or sMRI or vMRI) adj3 (brain* or cerebral or temporal or cortical or subcortical or hippocamp* or parahippocamp* or frontal or parietal or temporoparietal or frontoparietal or cerebell* or intracranial or gr#y matter or white matter or ventricular or orbitofrontal or bifrontal or cortices or diencephalon or pallidum or amygdala* or frontotemporal or thalamus or subthalamus or neuroanatomical or third ventricle* or midbrain or parenchymal or thalami? or subthalami? or basal ganglia or prefrontal or temporopolar or angular gyrus or postcentral gyrus or precuneus or cingulate gyrus or gyrus rectus or anterior cingulate or posterior cingulate or corpus callosum or insula or claustrum or caudate or frontal gyrus or precentral gyrus or supplementary motor area or paracentral lobule or postcentral gyrus or pons or medulla oblongata or angular gyrus or supramarginal gyrus or posterior cingulum or motor cortex or fusiform gyrus or occipital or frontal cortex or nucleus accumbens or globus pallidus or putamen or primary cortex)).mp. |
| 13 | 6 and 12 |
| 14 | ((MRI or MR imag* or MR scan* or MR imag* or Magnetic resonance imag* or Structural MRI or functional MRI or NMR imag* or T1 weighted imag* or T1-weighted MRI? or tensor-based morphometry or MRI measurement* or morphometric MRI or MR? biomarker* or Morphologic MRI or fMRI or sMRI or vMRI) adj6 (atroph* or degenerati* or neurodegenerat* or alteration* or changes or volume loss or "loss of volume" or microstructural degradation) adj7 (brain* or cerebral or temporal or cortical or subcortical or hippocamp* or parahippocamp* or frontal or parietal or temporoparietal or frontoparietal or cerebell* or intracranial or gr#y matter or white matter or ventricular or orbitofrontal or bifrontal or cortices or diencephalon or pallidum or amygdala* or frontotemporal or thalamus or subthalamus or neuroanatomical or third ventricle* or midbrain or parenchymal or thalami? or subthalami? or basal ganglia or prefrontal or temporopolar or angular gyrus or postcentral gyrus or precuneus or cingulate gyrus or gyrus rectus or anterior cingulate or posterior cingulate or corpus callosum or insula or claustrum or caudate or frontal gyrus or precentral gyrus or supplementary motor area or paracentral lobule or postcentral gyrus or pons or medulla oblongata or angular gyrus or supramarginal gyrus or posterior cingulum or motor cortex or fusiform gyrus or occipital or frontal cortex or nucleus accumbens or globus pallidus or putamen or primary cortex)).mp. |
| 15 | 6 and 14 |
| 16 | ((Volumetric brain MRI or MRI brain volumetry or brain MRI or Brain Magnetic Resonance or Brain MR) and (brain* or cerebral or temporal or cortical or subcortical or hippocamp* or parahippocamp* or frontal or parietal or temporoparietal or frontoparietal or cerebell* or intracranial or gr#y matter or white matter or ventricular or orbitofrontal or bifrontal or cortices or diencephalon or pallidum or amygdala* or frontotemporal or thalamus or subthalamus or neuroanatomical or third ventricle* or midbrain or parenchymal or thalami? or subthalami? or basal ganglia or prefrontal or temporopolar or angular gyrus or postcentral gyrus or precuneus or cingulate gyrus or gyrus rectus or anterior cingulate or posterior cingulate or corpus callosum or insula or claustrum or caudate or frontal gyrus or precentral gyrus or supplementary motor area or paracentral lobule or postcentral gyrus or pons or medulla oblongata or angular gyrus or supramarginal gyrus or posterior cingulum or motor cortex or fusiform gyrus or occipital or frontal cortex or nucleus accumbens or globus pallidus or putamen or primary cortex)).mp. |
| 17 | 6 and 16 |
| 18 | 9 or 11 or 13 or 15 or 17 |
| 19 | ((gr#y matter or white matter) adj (loss or damage* or degradation) adj7 (MRI? or Magnetic Resonance Imag* or MR imag* or MR scan* or Structural MRI or functional MRI or NMR imag* or T1 weighted imag* or T1-weighted MRI? or tensor-based morphometry or T1WI MRI or fMRI or FMRIB or sMRI or MRI assessment* or MRI stud* or MRI analys* or MRI-based or MRI measure* or MRI marker* or vMRI or Magnetic Resonance Imag* or morphometric MRI or MR? Biomarker*)).mp. |
| 20 | 6 and 19 |
| 21 | 18 or 20 |
| 22 | ((MRI? or Magnetic Resonance Imag* or MR imag* or MR scan* or Structural MRI or functional MRI or NMR imag* or T1 weighted imag* or T1-weighted MRI? or tensor-based morphometry or T1WI MRI or fMRI or FMRIB or sMRI or MRI assessment* or MRI stud* or MRI analys* or MRI-based or MRI measure* or MRI marker* or vMRI or Magnetic Resonance Imag* or morphometric MRI or MR? Biomarker*) and ((hypometabolism or metabolic or metabolism or hypermetabolism) adj7 (brain* or cerebral or temporal or cortical or subcortical or hippocamp* or parahippocamp* or frontal or parietal or temporoparietal or frontoparietal or cerebell* or intracranial or gr#y matter or white matter or ventricular or orbitofrontal or bifrontal or cortices or diencephalon or pallidum or amygdala* or frontotemporal or thalamus or subthalamus or neuroanatomical or third ventricle* or midbrain or parenchymal or thalami? or subthalami? or basal ganglia or prefrontal or temporopolar or angular gyrus or postcentral gyrus or precuneus or cingulate gyrus or gyrus rectus or anterior cingulate or posterior cingulate or corpus callosum or insula or claustrum or caudate or frontal gyrus or precentral gyrus or supplementary motor area or paracentral lobule or postcentral gyrus or pons or medulla oblongata or angular gyrus or supramarginal gyrus or posterior cingulum or motor cortex or fusiform gyrus or occipital or frontal cortex or nucleus accumbens or globus pallidus or putamen or primary cortex))).mp. |
| 23 | 6 and 22 |
| 24 | 21 or 23 |
| 25 | ((bvFTD or fvFTD or tvFTD or svPPA or nfvPPA or lpPPA or lvPPA or PPA-L or PNFA or SemD or PPA-G or nfPPA or PPA-S or PSP-RS or PSPS or PSP-P or PAGF or PSP-PGF or PSP-SL or PSP-F or PSP-bvFTD or PSP-PNFA or PSP-C or vPSP or FTD-MND or ALS-MND or ALS-FTD or ALS?FTD or CBS-CBD or CBS-PSP or PSP-CBS or PSP-PAGF or ALS or CBS or CBD or PSP or FTLD or MND or sMND or PPA or LPA or PNFA or FTD or FTLD-TDP or FTLD-tau or sFTLD or gFTLD or sMND or C9FTLD) adj8 (hypometabolism or Hypermetabolism or metabolic or metabolism)).mp. |
| 26 | ((bvFTD or fvFTD or tvFTD or svPPA or nfvPPA or lpPPA or lvPPA or PPA-L or PNFA or SemD or PPA-G or nfPPA or PPA-S or PSP-RS or PSPS or PSP-P or PAGF or PSP-PGF or PSP-SL or PSP-F or PSP-bvFTD or PSP-PNFA or PSP-C or vPSP or FTD-MND or ALS-MND or ALS-FTD or ALS?FTD or CBS-CBD or CBS-PSP or PSP-CBS or PSP-PAGF or ALS or CBS or CBD or PSP or FTLD or MND or sMND or PPA or LPA or PNFA or FTD or FTLD-TDP or FTLD-tau or sFTLD or gFTLD or sMND or C9FTLD) adj6 (atrophy or degeneration or volume* or changes in structure or lower GM density or altered functional connectivity or WM alteration* or WM loss or GM loss or GM volume* or GM atrophy or GM change* or structural change* or microstructural degradation* or microstructural alteration* or macrostructural atrophy)).mp. |
| 27 | 25 or 26 |
| 28 | 7 and 27 |
| 29 | 24 or 28 |
| 30 | exp animals/ not humans.sh. |
| 31 | 29 not 30 |
| 32 | limit 31 to english language |
| 33 | limit 31 to (case reports or clinical trial, veterinary or comment or editorial or letter or news or newspaper article or observational study, veterinary or randomized controlled trial, veterinary or video-audio media or webcast) |
| 34 | 32 not 33 |
| 35 | Fluorodeoxyglucose F18/ and (positron-emission tomography/ or positron emission tomography computed tomography/) |
| 36 | ((FDG adj5 PET) or (FDG? positron emission tomograph* or FDGPET)).mp. |
| 37 | ((Fluorodeoxyglucose or "Flu-deoxyglucose" or fludeoxyglucose or "Flu-deoxy-glucose" or "Fluoro Deoxy glucose" or 18F?FDG or "2-[18F]FDG" or "18F-FDG" or 18fdg or "18?F?Fluorodeoxyglucose" or 18Fluorodeoxyglucose or "18F?fluorodeoxyglucose" or "18?F?fluoro-2-deoxy" or "18F? Fluoro-2-Deoxy-D-Glucose" or "2-deoxy-2-[18F]fluoro-D-glucose" or "2 fluoro 2 deoxy d glucose" or "2 fluoro 2 deoxyglucose" or "2 deoxy 2 fluoro d glucose f 18" or "2 deoxy 2 fluoro dextro glucose f 18" or "2 deoxy 2 fluoro glucose f 18" or "2 deoxy 2 fluoroglucose f 18" or "2 deoxy d glucose f 18" or "2 deoxy dextro glucose f 18" or "deoxyfluoroglucose f 18" or "deoxyglucose f 18" or "Fluor-18-FDG" or "fluoro 2 deoxy d glucose f 18" or "fluoro 2 deoxyglucose f 18" or "fluorodeoxy d glucose f 18" or "2 deoxy 2 fluoro d glucose f18" or "2 deoxy 2 fluoro dextro glucose f18" or "2 deoxy 2 fluoro glucose f18" or "2 deoxy 2 fluoroglucose f18" or "2 deoxy d glucose f18" or "2 deoxy dextro glucose f18" or "deoxyfluoroglucose f18" or "deoxyglucose f18" or "fluoro 2 deoxy d glucose f18" or "fluoro 2 deoxyglucose f18" or "fluorodeoxy d glucose f18") and (positron emission tomograph* or PET)).mp. |
| 38 | 35 or 36 or 37 |
| 39 | 6 and 38 |
| 40 | ((bvFTD or fvFTD or tvFTD or svPPA or nfvPPA or lpPPA or lvPPA or PPA-L or PNFA or SemD or PPA-G or nfPPA or PPA-S or PSP-RS or PSPS or PSP-P or PAGF or PSP-PGF or PSP-SL or PSP-F or PSP-bvFTD or PSP-PNFA or PSP-C or vPSP or FTD-MND or ALS-MND or ALS-FTD or ALS?FTD or CBS-CBD or CBS-PSP or PSP-CBS or PSP-PAGF or ALS or CBS or CBD or PSP or FTLD or MND or sMND or PPA or LPA or PNFA or FTD or FTLD-TDP or FTLD-tau or sFTLD or gFTLD or sMND or C9FTLD) adj8 (hypometabolism or Hypermetabolism or metabolic or metabolism)).mp. |
| 41 | 38 and 40 |
| 42 | ((bvFTD or fvFTD or tvFTD or svPPA or nfvPPA or lpPPA or lvPPA or PPA-L or PNFA or SemD or PPA-G or nfPPA or PPA-S or PSP-RS or PSPS or PSP-P or PAGF or PSP-PGF or PSP-SL or PSP-F or PSP-bvFTD or PSP-PNFA or PSP-C or vPSP or FTD-MND or ALS-MND or ALS-FTD or ALS?FTD or CBS-CBD or CBS-PSP or PSP-CBS or PSP-PAGF or ALS or CBS or CBD or PSP or FTLD or MND or sMND or PPA or LPA or PNFA or FTD or FTLD-TDP or FTLD-tau or sFTLD or gFTLD or sMND or C9FTLD) adj6 (atrophy or degeneration or volume* or changes in structure or lower GM density or altered functional connectivity or WM alteration* or WM loss or GM loss or GM volume* or GM atrophy or GM change* or structural change* or microstructural degradation* or microstructural alteration* or macrostructural atrophy)).mp. |
| 43 | 38 and 42 |
| 44 | ((bvFTD or fvFTD or tvFTD or svPPA or nfvPPA or lpPPA or lvPPA or PPA-L or PNFA or SemD or PPA-G or nfPPA or PPA-S or PSP-RS or PSPS or PSP-P or PAGF or PSP-PGF or PSP-SL or PSP-F or PSP-bvFTD or PSP-PNFA or PSP-C or vPSP or FTD-MND or ALS-MND or ALS-FTD or ALS?FTD or CBS-CBD or CBS-PSP or PSP-CBS or PSP-PAGF or ALS or CBS or CBD or PSP or FTLD or MND or sMND or PPA or LPA or PNFA or FTD or FTLD-TDP or FTLD-tau or sFTLD or gFTLD or sMND or C9FTLD) adj10 ((Fluorodeoxyglucose or "Flu-deoxyglucose" or fludeoxyglucose or "Flu-deoxy-glucose" or "Fluoro Deoxy glucose" or 18F?FDG or "2-[18F]FDG" or "18F-FDG" or 18fdg or "18?F?Fluorodeoxyglucose" or 18Fluorodeoxyglucose or "18F?fluorodeoxyglucose" or "18?F?fluoro-2-deoxy" or "18F? Fluoro-2-Deoxy-D-Glucose" or "2-deoxy-2-[18F]fluoro-D-glucose" or "2 fluoro 2 deoxy d glucose" or "2 fluoro 2 deoxyglucose" or "2 deoxy 2 fluoro d glucose f 18" or "2 deoxy 2 fluoro dextro glucose f 18" or "2 deoxy 2 fluoro glucose f 18" or "2 deoxy 2 fluoroglucose f 18" or "2 deoxy d glucose f 18" or "2 deoxy dextro glucose f 18" or "deoxyfluoroglucose f 18" or "deoxyglucose f 18" or "Fluor-18-FDG" or "fluoro 2 deoxy d glucose f 18" or "fluoro 2 deoxyglucose f 18" or "fluorodeoxy d glucose f 18" or "2 deoxy 2 fluoro d glucose f18" or "2 deoxy 2 fluoro dextro glucose f18" or "2 deoxy 2 fluoro glucose f18" or "2 deoxy 2 fluoroglucose f18" or "2 deoxy d glucose f18" or "2 deoxy dextro glucose f18" or "deoxyfluoroglucose f18" or "deoxyglucose f18" or "fluoro 2 deoxy d glucose f18" or "fluoro 2 deoxyglucose f18" or "fluorodeoxy d glucose f18") and (positron emission tomograph* or PET))).mp. |
| 45 | (((FDG adj5 PET) or (FDG? positron emission tomograph* or FDGPET)) adj12 (bvFTD or fvFTD or tvFTD or svPPA or nfvPPA or lpPPA or lvPPA or PPA-L or PNFA or SemD or PPA-G or nfPPA or PPA-S or PSP-RS or PSPS or PSP-P or PAGF or PSP-PGF or PSP-SL or PSP-F or PSP-bvFTD or PSP-PNFA or PSP-C or vPSP or FTD-MND or ALS-MND or ALS-FTD or ALS?FTD or CBS-CBD or CBS-PSP or PSP-CBS or PSP-PAGF or ALS or CBS or CBD or PSP or FTLD or MND or sMND or PPA or LPA or PNFA or FTD or FTLD-TDP or FTLD-tau or sFTLD or gFTLD or sMND or C9FTLD)).mp. |
| 46 | (bvFTD or fvFTD or tvFTD or svPPA or nfvPPA or lpPPA or lvPPA or PPA-L or PNFA or SemD or PPA-G or nfPPA or PPA-S or PSP-RS or PSPS or PSP-P or PAGF or PSP-PGF or PSP-SL or PSP-F or PSP-bvFTD or PSP-PNFA or PSP-C or vPSP or FTD-MND or ALS-MND or ALS-FTD or ALS?FTD or CBS-CBD or CBS-PSP or PSP-CBS or PSP-PAGF or ALS or CBS or CBD or PSP or FTLD or MND or sMND or PPA or LPA or PNFA or FTD or FTLD-TDP or FTLD-tau or sFTLD or gFTLD or sMND or C9FTLD).mp. and (Fluorodeoxyglucose F18/ and (positron-emission tomography/ or positron emission tomography computed tomography/)) |
| 47 | 39 or 41 or 43 or 44 or 45 or 46 |
| 48 | exp animals/ not humans.sh. |
| 49 | 47 not 48 |
| 50 | limit 49 to english language |
| 51 | limit 50 to (case reports or clinical trial, veterinary or comment or editorial or letter or news or newspaper article or observational study, veterinary or randomized controlled trial, veterinary or video-audio media or webcast) |
| 52 | 50 not 51 |
| 53 | 34 or 52 |
| 54 | ((voxel-based morphometry or voxel-wise comparison* or voxel-wise exploration* or voxel-wise analys?s or voxel-level analys?s or voxel-wise correlation* or voxel-based volumetry or voxelbased morphometry or voxelwise comparison* or voxelwise exploration* or voxelwise analys?s or voxelbased analys?s or voxelwise correlation* or voxelbased volumetry or atlas-based volumetry or voxel-by-voxel basis or automatic volumetry or volume-based morphometry or volumetric quantification* or volumetric assessment* or volumetric change* or volumetric reduction* or volumetric indices or volumetric alteration* or volumetrically identifiable alteration* or volumetric analys?s or volumetric measure* or neurodegeneration biomarker* or degeneration pattern* or morphologic alteration* or structural alteration* or structural change* or microstructural degradation or macrostructural atrophy or microstructural alteration) and (brain* or cerebral or temporal or cortical or subcortical or hippocamp* or parahippocamp* or frontal or parietal or temporoparietal or frontoparietal or cerebell* or intracranial or gr#y matter or white matter or ventricular or orbitofrontal or bifrontal or cortices or diencephalon or pallidum or amygdala* or frontotemporal or thalamus or subthalamus or neuroanatomical or third ventricle* or midbrain or parenchymal or thalami? or subthalami? or basal ganglia or prefrontal or temporopolar or angular gyrus or postcentral gyrus or precuneus or cingulate gyrus or gyrus rectus or anterior cingulate or posterior cingulate or corpus callosum or insula or claustrum or caudate or frontal gyrus or precentral gyrus or supplementary motor area or paracentral lobule or postcentral gyrus or pons or medulla oblongata or angular gyrus or supramarginal gyrus or posterior cingulum or motor cortex or fusiform gyrus or occipital or frontal cortex or nucleus accumbens or globus pallidus or putamen or primary cortex)).mp. |
| 55 | 6 and 7 and 54 |
| 56 | exp animals/ not humans.sh. |
| 57 | 55 not 56 |
| 58 | limit 57 to english language |
| 59 | limit 58 to (case reports or clinical trial, veterinary or comment or editorial or letter or news or newspaper article or observational study, veterinary or randomized controlled trial, veterinary or video-audio media or webcast) |
| 60 | 58 not 59 |
| 61 | 53 or 60 |
| 62 | Disease Models, Animal/ |
| 63 | (animal model* or mouse model*).mp. |
| 64 | (rat or rats or mouse or mice or swine or porcine or murine or sheep? or lamb or lambs or pig or pigs or piglets or rabbit or rabbits or cat or cats or dog or dogs or cattle or monkey or monkeys or trout or marmoset* or hamster* or primate* or rodent* or Anserine or Aquiline or Assinine or Bovine or Canine or Cervine or Equine or Elaphine or Feline or Hircine or Leporine or Lupine or Murine or Ovine or Porcine or Rusine or Serpentine or Simian or Ursine or Vulpine or Guinea pig or guinea fowl).mp. |
| 65 | 62 or 63 or 64 |
| 66 | 61 not 65 |
| 67 | (case report? or comment or editorial or letter or newspaper or veterinary or video-audio media or webcast).ti. |
| **68** | **66 not 67** |
| 69 | (Imaging Signatures of Molecular Pathology in Behavioral Variant Frontotemporal Dementia or Different Patterns of Magnetic resonance imaging Atrophy for Frontotemporal Lobar Degeneration Syndromes or "Structural MRI Signatures in Genetic Presentations of the Frontotemporal Dementia/Motor Neuron Disease Spectrum" or Different FDG-PET metabolic patterns at single-subject level in the behavioral variant of fronto-temporal dementia or (Comparing brain structural MRI and metabolic FDG-PET changes in patients with ALS-FTD) or (Brain atrophy over time in genetic and sporadic frontotemporal dementia: a study of 198 serial magnetic resonance images) or Patterns of Frontal Lobe Atrophy in Frontotemporal Dementia: A Volumetric MRI Study or (MRI Signatures of Brain Macrostructural Atrophy and Microstructural Degradation in Frontotemporal Lobar Degeneration Subtypes) or Anatomical MRI staging of frontotemporal dementia variants).ti. |
| 70 | 68 and 69 |

**DATABASE 2: EMBASE**

Embase <1947 to 2024 August 28>

| **#** | **Query** |
| --- | --- |
| 1 | frontotemporal dementia/ or frontal variant frontotemporal dementia/ or Pick presenile dementia/ or amyotrophic lateral sclerosis/ or corticobasal degeneration/ or motor neuron disease/ or progressive supranuclear palsy/ or primary progressive aphasia/ or progressive nonfluent aphasia/ or semantic dementia/ |
| 2 | RNA binding protein FUS/ or guanine nucleotide exchange C9orf72/ or TDP 43 proteinopathy/ or tau protein/ or tauopathy/ |
| 3 | (frontotemporal lobar degeneration or fronto temporal lobar degeneration or frontal dementia or frontal lobe dementia or Pick* complex or frontotemporal dementia or frontal-temporal dementia or fronto temporal dementia or behavio?ral variant FTD or frontal variant FTD or Pick* disease or Pick dementia or Pick syndrome or FTD-behavio?ral variant* or FTD-language variant* or amyotrophic lateral scleros?s or ALS dementia or motor neuron disease* or sporadic MND or motoneuron disease* or motor neurone disease* or motor system disease* or primary lateral scleros?s or corticobasal syndrome* or corticobasal degeneration or cortical basal degeneration or cortical basal ganglionic degeneration or corticobasal ganglionic degeneration or cortico basal ganglionic degeneration or primary progressive aphasia* or temporal variant FTD or PPA syndrome* or semantic aphasia* or logopenic variant PPA or logopenic progressive aphasia* or "progressive non-fluent aphasia*" or "Non-fluent progressive aphasia*" or nonfluent progressive aphasia* or progressive nonfluent aphasia* or "non-fluent primary progressive aphasia" or semantic dementia or "Non-fluent?agrammatic variant*" or Nonfluent?agrammatic variant* or semantic PPA or Semantic variant PPA or progressive supranuclear palsy or Steele-Olszewski-Richardson Syndrome or steele richardson olszewski syndrome or supranuclear progressive palsy or PSP-Richardson* syndrome or "PSP-pure akinesia with gait freezing" or PSP-Progressive gait freezing or "PSP-Speech and Language variant" or PSP-Speech?Language or PSP-Frontal or Frontal PSP or Progressive supranuclear ophthalmoplegia* or vertical supranuclear ophthalmoplegia* or supranuclear vertical gaze palsy or vertical supranuclear gaze palsy).mp. |
| 4 | (C9orf72 or MAPT or TDP43 or "TDP-43" or TARDBP43 or "TARDBP-43" or TAU protein or "fused in sarcoma" or FUS gene* or FUS protein or FUS RNA binding protein or "protein FUS" or "TAR DNA binding protein 43" or "C9 open reading frame 72" or "chromosome 9 open reading frame 72" or Tauopath* or "transactive response DNA binding protein 43").mp. |
| 5 | (bvFTD or fvFTD or tvFTD or svPPA or nfvPPA or lpPPA or lvPPA or PPA-L or PNFA or SemD or PPA-G or nfPPA or PPA-S or PSP-RS or PSPS or PSP-P or PAGF or PSP-PGF or PSP-SL or PSP-F or PSP-bvFTD or PSP-PNFA or PSP-C or vPSP or FTD-MND or ALS-MND or ALS-FTD or ALS?FTD or CBS-CBD or CBS-PSP or PSP-CBS or PSP-PAGF or FTLD-TDP or FTLD-tau or sFTLD or gFTLD or sMND or C9FTLD).mp. |
| 6 | 1 or 2 or 3 or 4 or 5 |
| 7 | nuclear magnetic resonance imaging/ or T1 weighted imaging/ or functional magnetic resonance imaging/ |
| 8 | ((volumetric* or volumetry or volumes or structural change* or structural* alterat* or "change* in structure" or microstructural degradation or macrostructural atrophy) and (brain* or cerebral or temporal or cortical or subcortical or hippocamp* or parahippocamp* or frontal or parietal or temporoparietal or frontoparietal or cerebell* or intracranial or gr#y matter or white matter or ventricular or orbitofrontal or bifrontal or cortices or diencephalon or pallidum or amygdala* or frontotemporal or thalamus or subthalamus or neuroanatomical or third ventricle* or midbrain or parenchymal or thalami? or subthalami? or basal ganglia or prefrontal or temporopolar or angular gyrus or postcentral gyrus or precuneus or cingulate gyrus or gyrus rectus or anterior cingulate or posterior cingulate or corpus callosum or insula or claustrum or caudate or frontal gyrus or precentral gyrus or supplementary motor area or paracentral lobule or postcentral gyrus or pons or medulla oblongata or angular gyrus or supramarginal gyrus or posterior cingulum or motor cortex or fusiform gyrus or occipital or frontal cortex or nucleus accumbens or globus pallidus or putamen or primary cortex)).mp. |
| 9 | 6 and 7 and 8 |
| 10 | (((MRI? or Magnetic Resonance Imag* or MR imag* or MR scan* or Structural MRI or functional MRI or NMR imag* or T1 weighted imag* or T1-weighted MRI? or tensor-based morphometry or T1WI MRI or fMRI or FMRIB or sMRI or MRI assessment* or MRI stud* or MRI analys* or MRI-based or MRI measure* or MRI marker* or vMRI or Magnetic Resonance Imag* or morphometric MRI or MR? Biomarker*) adj7 (volumetric* or volumetry or volumes or structural change* or structural* alterat* or "change* in structure" or microstructural degradation or macrostructural atrophy)) and (brain* or cerebral or temporal or cortical or subcortical or hippocamp* or parahippocamp* or frontal or parietal or temporoparietal or frontoparietal or cerebell* or intracranial or gr#y matter or white matter or ventricular or orbitofrontal or bifrontal or cortices or diencephalon or pallidum or amygdala* or frontotemporal or thalamus or subthalamus or neuroanatomical or third ventricle* or midbrain or parenchymal or thalami? or subthalami? or basal ganglia or prefrontal or temporopolar or angular gyrus or postcentral gyrus or precuneus or cingulate gyrus or gyrus rectus or anterior cingulate or posterior cingulate or corpus callosum or insula or claustrum or caudate or frontal gyrus or precentral gyrus or supplementary motor area or paracentral lobule or postcentral gyrus or pons or medulla oblongata or angular gyrus or supramarginal gyrus or posterior cingulum or motor cortex or fusiform gyrus or occipital or frontal cortex or nucleus accumbens or globus pallidus or putamen or primary cortex)).mp. |
| 11 | 6 and 10 |
| 12 | ((MR scan* or MR imag* or Magnetic resonance imag* or Structural MRI or functional MRI or NMR imag* or T1 weighted imag* or T1-weighted MRI? or tensor-based morphometry or morphometric MRI or MR? Biomarker* or MRI measure* or fMRI or sMRI or vMRI) adj3 (brain* or cerebral or temporal or cortical or subcortical or hippocamp* or parahippocamp* or frontal or parietal or temporoparietal or frontoparietal or cerebell* or intracranial or gr#y matter or white matter or ventricular or orbitofrontal or bifrontal or cortices or diencephalon or pallidum or amygdala* or frontotemporal or thalamus or subthalamus or neuroanatomical or third ventricle* or midbrain or parenchymal or thalami? or subthalami? or basal ganglia or prefrontal or temporopolar or angular gyrus or postcentral gyrus or precuneus or cingulate gyrus or gyrus rectus or anterior cingulate or posterior cingulate or corpus callosum or insula or claustrum or caudate or frontal gyrus or precentral gyrus or supplementary motor area or paracentral lobule or postcentral gyrus or pons or medulla oblongata or angular gyrus or supramarginal gyrus or posterior cingulum or motor cortex or fusiform gyrus or occipital or frontal cortex or nucleus accumbens or globus pallidus or putamen or primary cortex)).mp. |
| 13 | 6 and 12 |
| 14 | ((MRI or MR imag* or MR scan* or MR imag* or Magnetic resonance imag* or Structural MRI or functional MRI or NMR imag* or T1 weighted imag* or T1-weighted MRI? or tensor-based morphometry or MRI measurement* or morphometric MRI or MR? biomarker* or Morphologic MRI or fMRI or sMRI or vMRI) adj6 (atroph* or degenerati* or neurodegenerat* or alteration* or changes or volume loss or "loss of volume" or microstructural degradation) adj7 (brain* or cerebral or temporal or cortical or subcortical or hippocamp* or parahippocamp* or frontal or parietal or temporoparietal or frontoparietal or cerebell* or intracranial or gr#y matter or white matter or ventricular or orbitofrontal or bifrontal or cortices or diencephalon or pallidum or amygdala* or frontotemporal or thalamus or subthalamus or neuroanatomical or third ventricle* or midbrain or parenchymal or thalami? or subthalami? or basal ganglia or prefrontal or temporopolar or angular gyrus or postcentral gyrus or precuneus or cingulate gyrus or gyrus rectus or anterior cingulate or posterior cingulate or corpus callosum or insula or claustrum or caudate or frontal gyrus or precentral gyrus or supplementary motor area or paracentral lobule or postcentral gyrus or pons or medulla oblongata or angular gyrus or supramarginal gyrus or posterior cingulum or motor cortex or fusiform gyrus or occipital or frontal cortex or nucleus accumbens or globus pallidus or putamen or primary cortex)).mp. |
| 15 | 6 and 14 |
| 16 | ((Volumetric brain MRI or MRI brain volumetry or brain MRI or Brain Magnetic Resonance or Brain MR) and (brain* or cerebral or temporal or cortical or subcortical or hippocamp* or parahippocamp* or frontal or parietal or temporoparietal or frontoparietal or cerebell* or intracranial or gr#y matter or white matter or ventricular or orbitofrontal or bifrontal or cortices or diencephalon or pallidum or amygdala* or frontotemporal or thalamus or subthalamus or neuroanatomical or third ventricle* or midbrain or parenchymal or thalami? or subthalami? or basal ganglia or prefrontal or temporopolar or angular gyrus or postcentral gyrus or precuneus or cingulate gyrus or gyrus rectus or anterior cingulate or posterior cingulate or corpus callosum or insula or claustrum or caudate or frontal gyrus or precentral gyrus or supplementary motor area or paracentral lobule or postcentral gyrus or pons or medulla oblongata or angular gyrus or supramarginal gyrus or posterior cingulum or motor cortex or fusiform gyrus or occipital or frontal cortex or nucleus accumbens or globus pallidus or putamen or primary cortex)).mp. |
| 17 | 6 and 16 |
| 18 | 9 or 11 or 13 or 15 or 17 |
| 19 | ((gr#y matter or white matter) adj (loss or damage* or degradation) adj7 (MRI? or Magnetic Resonance Imag* or MR imag* or MR scan* or Structural MRI or functional MRI or NMR imag* or T1 weighted imag* or T1-weighted MRI? or tensor-based morphometry or T1WI MRI or fMRI or FMRIB or sMRI or MRI assessment* or MRI stud* or MRI analys* or MRI-based or MRI measure* or MRI marker* or vMRI or Magnetic Resonance Imag* or morphometric MRI or MR? Biomarker*)).mp. |
| 20 | 6 and 19 |
| 21 | 18 or 20 |
| 22 | ((MRI? or Magnetic Resonance Imag* or MR imag* or MR scan* or Structural MRI or functional MRI or NMR imag* or T1 weighted imag* or T1-weighted MRI? or tensor-based morphometry or T1WI MRI or fMRI or FMRIB or sMRI or MRI assessment* or MRI stud* or MRI analys* or MRI-based or MRI measure* or MRI marker* or vMRI or Magnetic Resonance Imag* or morphometric MRI or MR? Biomarker*) and ((hypometabolism or metabolic or metabolism or hypermetabolism) adj7 (brain* or cerebral or temporal or cortical or subcortical or hippocamp* or parahippocamp* or frontal or parietal or temporoparietal or frontoparietal or cerebell* or intracranial or gr#y matter or white matter or ventricular or orbitofrontal or bifrontal or cortices or diencephalon or pallidum or amygdala* or frontotemporal or thalamus or subthalamus or neuroanatomical or third ventricle* or midbrain or parenchymal or thalami? or subthalami? or basal ganglia or prefrontal or temporopolar or angular gyrus or postcentral gyrus or precuneus or cingulate gyrus or gyrus rectus or anterior cingulate or posterior cingulate or corpus callosum or insula or claustrum or caudate or frontal gyrus or precentral gyrus or supplementary motor area or paracentral lobule or postcentral gyrus or pons or medulla oblongata or angular gyrus or supramarginal gyrus or posterior cingulum or motor cortex or fusiform gyrus or occipital or frontal cortex or nucleus accumbens or globus pallidus or putamen or primary cortex))).mp. |
| 23 | 6 and 22 |
| 24 | 21 or 23 |
| 25 | ((bvFTD or fvFTD or tvFTD or svPPA or nfvPPA or lpPPA or lvPPA or PPA-L or PNFA or SemD or PPA-G or nfPPA or PPA-S or PSP-RS or PSPS or PSP-P or PAGF or PSP-PGF or PSP-SL or PSP-F or PSP-bvFTD or PSP-PNFA or PSP-C or vPSP or FTD-MND or ALS-MND or ALS-FTD or ALS?FTD or CBS-CBD or CBS-PSP or PSP-CBS or PSP-PAGF or ALS or CBS or CBD or PSP or FTLD or MND or sMND or PPA or LPA or PNFA or FTD or FTLD-TDP or FTLD-tau or sFTLD or gFTLD or sMND or C9FTLD) adj8 (hypometabolism or Hypermetabolism or metabolic or metabolism)).mp. |
| 26 | ((bvFTD or fvFTD or tvFTD or svPPA or nfvPPA or lpPPA or lvPPA or PPA-L or PNFA or SemD or PPA-G or nfPPA or PPA-S or PSP-RS or PSPS or PSP-P or PAGF or PSP-PGF or PSP-SL or PSP-F or PSP-bvFTD or PSP-PNFA or PSP-C or vPSP or FTD-MND or ALS-MND or ALS-FTD or ALS?FTD or CBS-CBD or CBS-PSP or PSP-CBS or PSP-PAGF or ALS or CBS or CBD or PSP or FTLD or MND or sMND or PPA or LPA or PNFA or FTD or FTLD-TDP or FTLD-tau or sFTLD or gFTLD or sMND or C9FTLD) adj6 (atrophy or degeneration or volume* or changes in structure or lower GM density or altered functional connectivity or WM alteration* or WM loss or GM loss or GM volume* or GM atrophy or GM change* or structural change* or microstructural degradation* or microstructural alteration* or macrostructural atrophy)).mp. |
| 27 | 25 or 26 |
| 28 | 7 and 27 |
| 29 | 24 or 28 |
| 30 | (exp animal/ or nonhuman/ or exp invertebrate/ or animal.hw.) not exp human/ |
| 31 | 29 not 30 |
| 32 | limit 31 to english language |
| 33 | limit 32 to (conference abstract or editorial or letter or note) |
| 34 | 32 not 33 |
| 35 | fluorodeoxyglucose f 18/ and (positron emission tomography/ or positron emission tomography-computed tomography/) |
| 36 | ((FDG adj5 PET) or (FDG? positron emission tomograph* or FDGPET)).mp. |
| 37 | ((Fluorodeoxyglucose or "Flu-deoxyglucose" or fludeoxyglucose or "Flu-deoxy-glucose" or "Fluoro Deoxy glucose" or 18F?FDG or "2-[18F]FDG" or "18F-FDG" or 18fdg or "18?F?Fluorodeoxyglucose" or 18Fluorodeoxyglucose or "18F?fluorodeoxyglucose" or "18?F?fluoro-2-deoxy" or "18F? Fluoro-2-Deoxy-D-Glucose" or "2-deoxy-2-[18F]fluoro-D-glucose" or "2 fluoro 2 deoxy d glucose" or "2 fluoro 2 deoxyglucose" or "2 deoxy 2 fluoro d glucose f 18" or "2 deoxy 2 fluoro dextro glucose f 18" or "2 deoxy 2 fluoro glucose f 18" or "2 deoxy 2 fluoroglucose f 18" or "2 deoxy d glucose f 18" or "2 deoxy dextro glucose f 18" or "deoxyfluoroglucose f 18" or "deoxyglucose f 18" or "Fluor-18-FDG" or "fluoro 2 deoxy d glucose f 18" or "fluoro 2 deoxyglucose f 18" or "fluorodeoxy d glucose f 18" or "2 deoxy 2 fluoro d glucose f18" or "2 deoxy 2 fluoro dextro glucose f18" or "2 deoxy 2 fluoro glucose f18" or "2 deoxy 2 fluoroglucose f18" or "2 deoxy d glucose f18" or "2 deoxy dextro glucose f18" or "deoxyfluoroglucose f18" or "deoxyglucose f18" or "fluoro 2 deoxy d glucose f18" or "fluoro 2 deoxyglucose f18" or "fluorodeoxy d glucose f18") and (positron emission tomograph* or PET)).mp. |
| 38 | 35 or 36 or 37 |
| 39 | 6 and 38 |
| 40 | ((bvFTD or fvFTD or tvFTD or svPPA or nfvPPA or lpPPA or lvPPA or PPA-L or PNFA or SemD or PPA-G or nfPPA or PPA-S or PSP-RS or PSPS or PSP-P or PAGF or PSP-PGF or PSP-SL or PSP-F or PSP-bvFTD or PSP-PNFA or PSP-C or vPSP or FTD-MND or ALS-MND or ALS-FTD or ALS?FTD or CBS-CBD or CBS-PSP or PSP-CBS or PSP-PAGF or ALS or CBS or CBD or PSP or FTLD or MND or sMND or PPA or LPA or PNFA or FTD or FTLD-TDP or FTLD-tau or sFTLD or gFTLD or sMND or C9FTLD) adj8 (hypometabolism or Hypermetabolism or metabolic or metabolism)).mp. |
| 41 | 38 and 40 |
| 42 | ((bvFTD or fvFTD or tvFTD or svPPA or nfvPPA or lpPPA or lvPPA or PPA-L or PNFA or SemD or PPA-G or nfPPA or PPA-S or PSP-RS or PSPS or PSP-P or PAGF or PSP-PGF or PSP-SL or PSP-F or PSP-bvFTD or PSP-PNFA or PSP-C or vPSP or FTD-MND or ALS-MND or ALS-FTD or ALS?FTD or CBS-CBD or CBS-PSP or PSP-CBS or PSP-PAGF or ALS or CBS or CBD or PSP or FTLD or MND or sMND or PPA or LPA or PNFA or FTD or FTLD-TDP or FTLD-tau or sFTLD or gFTLD or sMND or C9FTLD) adj6 (atrophy or degeneration or volume* or changes in structure or lower GM density or altered functional connectivity or WM alteration* or WM loss or GM loss or GM volume* or GM atrophy or GM change* or structural change* or microstructural degradation* or microstructural alteration* or macrostructural atrophy)).mp. |
| 43 | 38 and 42 |
| 44 | ((bvFTD or fvFTD or tvFTD or svPPA or nfvPPA or lpPPA or lvPPA or PPA-L or PNFA or SemD or PPA-G or nfPPA or PPA-S or PSP-RS or PSPS or PSP-P or PAGF or PSP-PGF or PSP-SL or PSP-F or PSP-bvFTD or PSP-PNFA or PSP-C or vPSP or FTD-MND or ALS-MND or ALS-FTD or ALS?FTD or CBS-CBD or CBS-PSP or PSP-CBS or PSP-PAGF or ALS or CBS or CBD or PSP or FTLD or MND or sMND or PPA or LPA or PNFA or FTD or FTLD-TDP or FTLD-tau or sFTLD or gFTLD or sMND or C9FTLD) adj10 ((Fluorodeoxyglucose or "Flu-deoxyglucose" or fludeoxyglucose or "Flu-deoxy-glucose" or "Fluoro Deoxy glucose" or 18F?FDG or "2-[18F]FDG" or "18F-FDG" or 18fdg or "18?F?Fluorodeoxyglucose" or 18Fluorodeoxyglucose or "18F?fluorodeoxyglucose" or "18?F?fluoro-2-deoxy" or "18F? Fluoro-2-Deoxy-D-Glucose" or "2-deoxy-2-[18F]fluoro-D-glucose" or "2 fluoro 2 deoxy d glucose" or "2 fluoro 2 deoxyglucose" or "2 deoxy 2 fluoro d glucose f 18" or "2 deoxy 2 fluoro dextro glucose f 18" or "2 deoxy 2 fluoro glucose f 18" or "2 deoxy 2 fluoroglucose f 18" or "2 deoxy d glucose f 18" or "2 deoxy dextro glucose f 18" or "deoxyfluoroglucose f 18" or "deoxyglucose f 18" or "Fluor-18-FDG" or "fluoro 2 deoxy d glucose f 18" or "fluoro 2 deoxyglucose f 18" or "fluorodeoxy d glucose f 18" or "2 deoxy 2 fluoro d glucose f18" or "2 deoxy 2 fluoro dextro glucose f18" or "2 deoxy 2 fluoro glucose f18" or "2 deoxy 2 fluoroglucose f18" or "2 deoxy d glucose f18" or "2 deoxy dextro glucose f18" or "deoxyfluoroglucose f18" or "deoxyglucose f18" or "fluoro 2 deoxy d glucose f18" or "fluoro 2 deoxyglucose f18" or "fluorodeoxy d glucose f18") and (positron emission tomograph* or PET))).mp. |
| 45 | (((FDG adj5 PET) or (FDG? positron emission tomograph* or FDGPET)) adj12 (bvFTD or fvFTD or tvFTD or svPPA or nfvPPA or lpPPA or lvPPA or PPA-L or PNFA or SemD or PPA-G or nfPPA or PPA-S or PSP-RS or PSPS or PSP-P or PAGF or PSP-PGF or PSP-SL or PSP-F or PSP-bvFTD or PSP-PNFA or PSP-C or vPSP or FTD-MND or ALS-MND or ALS-FTD or ALS?FTD or CBS-CBD or CBS-PSP or PSP-CBS or PSP-PAGF or ALS or CBS or CBD or PSP or FTLD or MND or sMND or PPA or LPA or PNFA or FTD or FTLD-TDP or FTLD-tau or sFTLD or gFTLD or sMND or C9FTLD)).mp. |
| 46 | (bvFTD or fvFTD or tvFTD or svPPA or nfvPPA or lpPPA or lvPPA or PPA-L or PNFA or SemD or PPA-G or nfPPA or PPA-S or PSP-RS or PSPS or PSP-P or PAGF or PSP-PGF or PSP-SL or PSP-F or PSP-bvFTD or PSP-PNFA or PSP-C or vPSP or FTD-MND or ALS-MND or ALS-FTD or ALS?FTD or CBS-CBD or CBS-PSP or PSP-CBS or PSP-PAGF or ALS or CBS or CBD or PSP or FTLD or MND or sMND or PPA or LPA or PNFA or FTD or FTLD-TDP or FTLD-tau or sFTLD or gFTLD or sMND or C9FTLD).mp. and (Fluorodeoxyglucose F18/ and (positron-emission tomography/ or positron emission tomography computed tomography/)) |
| 47 | 39 or 41 or 43 or 44 or 45 or 46 |
| 48 | (exp animal/ or nonhuman/ or exp invertebrate/ or animal.hw.) not exp human/ |
| 49 | 47 not 48 |
| 50 | limit 49 to english language |
| 51 | limit 50 to (conference abstract or editorial or letter or note) |
| 52 | 50 not 51 |
| 53 | 34 or 52 |
| 54 | ((voxel-based morphometry or voxel-wise comparison* or voxel-wise exploration* or voxel-wise analys?s or voxel-level analys?s or voxel-wise correlation* or voxel-based volumetry or voxelbased morphometry or voxelwise comparison* or voxelwise exploration* or voxelwise analys?s or voxelbased analys?s or voxelwise correlation* or voxelbased volumetry or atlas-based volumetry or voxel-by-voxel basis or automatic volumetry or volume-based morphometry or volumetric quantification* or volumetric assessment* or volumetric change* or volumetric reduction* or volumetric indices or volumetric alteration* or volumetrically identifiable alteration* or volumetric analys?s or volumetric measure* or neurodegeneration biomarker* or degeneration pattern* or morphologic alteration* or structural alteration* or structural change* or microstructural degradation or macrostructural atrophy or microstructural alteration) and (brain* or cerebral or temporal or cortical or subcortical or hippocamp* or parahippocamp* or frontal or parietal or temporoparietal or frontoparietal or cerebell* or intracranial or gr#y matter or white matter or ventricular or orbitofrontal or bifrontal or cortices or diencephalon or pallidum or amygdala* or frontotemporal or thalamus or subthalamus or neuroanatomical or third ventricle* or midbrain or parenchymal or thalami? or subthalami? or basal ganglia or prefrontal or temporopolar or angular gyrus or postcentral gyrus or precuneus or cingulate gyrus or gyrus rectus or anterior cingulate or posterior cingulate or corpus callosum or insula or claustrum or caudate or frontal gyrus or precentral gyrus or supplementary motor area or paracentral lobule or postcentral gyrus or pons or medulla oblongata or angular gyrus or supramarginal gyrus or posterior cingulum or motor cortex or fusiform gyrus or occipital or frontal cortex or nucleus accumbens or globus pallidus or putamen or primary cortex)).mp. |
| 55 | 6 and 7 and 54 |
| 56 | (exp animal/ or nonhuman/ or exp invertebrate/ or animal.hw.) not exp human/ |
| 57 | 55 not 56 |
| 58 | limit 57 to english language |
| 59 | limit 58 to (conference abstract or editorial or letter or note) |
| 60 | 58 not 59 |
| 61 | 53 or 60 |
| 62 | disease model/ |
| 63 | exp animal experiment/ or exp animal model/ or exp experimental animal/ or exp transgenic animal/ or exp male animal/ or exp female animal/ or exp juvenile animal/ or chordata/ or vertebrate/ or tetrapod/ or exp fish/ or amniote/ or exp amphibia/ or exp reptile/ or exp sauropsid/ or therian/ or exp monotreme/ or exp marsupial/ or Euarchontoglires/ or exp Afrotheria/ or exp Boreoeutheria/ or exp Laurasiatheria/ or exp Xenarthra/ or primate/ or exp Dermoptera/ or exp Glires/ or exp Scandentia/ or Haplorhini/ or exp prosimian/ or simian/ or exp tarsiiform/ or Catarrhini/ or exp Platyrrhini/ or ape/ or exp Cercopithecidae/ or exp hylobatidae/ or exp chimpanzee/ or exp gorilla/ or exp orang utan/ or exp ephalopod/ |
| 64 | (rat or rats or mouse or mice or swine or porcine or murine or sheep? or lamb or lambs or pig or pigs or piglets or rabbit or rabbits or cat or cats or dog or dogs or cattle or monkey or monkeys or trout or marmoset* or hamster* or primate* or rodent* or Anserine or Aquiline or Assinine or Bovine or Canine or Cervine or Equine or Elaphine or Feline or Hircine or Leporine or Lupine or Murine or Ovine or Porcine or Rusine or Serpentine or Simian or Ursine or Vulpine or Guinea pig or guinea fowl).mp. |
| 65 | 62 or 63 or 64 |
| 66 | 61 not 65 |
| 67 | (case report? or comment or editorial or letter or newspaper or veterinary or video-audio media or webcast).ti. |
| 68 | 66 not 67 |

**DATABASE 3: WEB OF SCIENCE**

| **#** | **Query** |
| --- | --- |
| 1 | (“frontotemporal lobar degeneration” or “fronto temporal lobar degeneration” or “frontal dementia” or “frontal lobe dementia” or “Pick* complex” or “frontotemporal dementia” or “frontal-temporal dementia” or “fronto temporal dementia” or “behavio?ral variant FTD” or “frontal variant FTD” or “Pick* disease” or “Pick dementia” or “Pick syndrome” or “FTD-behavio?ral variant*” or “FTD-language variant*” or “amyotrophic lateral scleros?s” or “ALS dementia” or “motor neuron disease*” or “sporadic MND” or “motoneuron disease*” or “motor neurone disease*” or “motor system disease*” or “primary lateral scleros?s” or “corticobasal syndrome*” or “corticobasal degeneration” or “cortical basal degeneration” or “cortical basal ganglionic degeneration” or “corticobasal ganglionic degeneration” or “cortico basal ganglionic degeneration” or “primary progressive aphasia*” or “temporal variant FTD” or “PPA syndrome*” or “semantic aphasia*” or “logopenic variant PPA” or “logopenic progressive aphasia*” or "progressive non-fluent aphasia*" or "Non-fluent progressive aphasia*" or “nonfluent progressive aphasia*” or “progressive nonfluent aphasia*” or "non-fluent primary progressive aphasia" or “semantic dementia” or "Non-fluent?agrammatic variant*" or “Nonfluent?agrammatic variant*” or “semantic PPA” or “Semantic variant PPA” or “progressive supranuclear palsy” or “Steele-Olszewski-Richardson Syndrome” or “steele richardson olszewski syndrome” or “supranuclear progressive palsy” or “PSP-Richardson* syndrome” or "PSP-pure akinesia with gait freezing" or “PSP-Progressive gait freezing” or "PSP-Speech and Language variant" or “PSP-Speech?Language” or “PSP-Frontal” or “Frontal PSP” or “Progressive supranuclear ophthalmoplegia*” or “vertical supranuclear ophthalmoplegia*” or “supranuclear vertical gaze palsy” or “vertical supranuclear gaze palsy”) (Topic) or (C9orf72 or MAPT or TDP43 or "TDP-43" or TARDBP43 or "TARDBP-43" or “TAU protein” or "fused in sarcoma" or “FUS gene*” or “FUS protein” or “FUS RNA binding protein” or "protein FUS" or "TAR DNA binding protein 43" or "C9 open reading frame 72" or "chromosome 9 open reading frame 72" or Tauopath* or "transactive response DNA binding protein 43") (Topic) or (bvFTD or fvFTD or tvFTD or svPPA or nfvPPA or lpPPA or lvPPA or “PPA-L” or PNFA or SemD or “PPA-G” or nfPPA or “PPA-S” or “PSP-RS” or PSPS or “PSP-P” or PAGF or “PSP-PGF” or “PSP-SL” or “PSP-F” or “PSP-bvFTD” or “PSP-PNFA” or “PSP-C” or vPSP or “FTD-MND” or “ALS-MND” or “ALS-FTD” or ALS?FTD or “CBS-CBD” or “CBS-PSP” or “PSP-CBS” or “PSP-PAGF” or “FTLD-TDP” or “FTLD-tau” or sFTLD or gFTLD or sMND or C9FTLD) (Topic) |
| 2 | ((MRI? or “Magnetic Resonance Imag*” or “MR imag*” or “MR scan*” or “Structural MRI” or “functional MRI” or “NMR imag*” or “T1 weighted imag*” or “T1-weighted MRI?” or “tensor-based morphometry” or “T1WI MRI” or fMRI or FMRIB or sMRI or “MRI assessment*” or “MRI stud*” or “MRI analys*” or “MRI-based” or “MRI measure*” or “MRI marker*” or vMRI or “Magnetic Resonance Imag*” or “morphometric MRI” or “MR? Biomarker*”) NEAR/6 (volumetric* or volumetry or volumes or “structural change*” or “structural* alterat*” or "change* in structure" or “microstructural degradation” or “macrostructural atrophy”)) (Topic) and (brain* or cerebral or temporal or cortical or subcortical or hippocamp* or parahippocamp* or frontal or parietal or temporoparietal or frontoparietal or cerebell* or intracranial or “gr?y matter” or “white matter” or ventricular or orbitofrontal or bifrontal or cortices or diencephalon or pallidum or amygdala* or frontotemporal or thalamus or subthalamus or neuroanatomical or “third ventricle*” or midbrain or parenchymal or thalami? or subthalami? or “basal ganglia” or prefrontal or temporopolar or “angular gyrus” or “postcentral gyrus” or precuneus or “cingulate gyrus” or “gyrus rectus” or “anterior cingulate” or “posterior cingulate” or “corpus callosum” or insula or claustrum or caudate or “frontal gyrus” or “precentral gyrus” or “supplementary motor area” or “paracentral lobule” or “postcentral gyrus” or pons or “medulla oblongata” or “angular gyrus” or “supramarginal gyrus” or “posterior cingulum” or “motor cortex” or “fusiform gyrus” or occipital or “frontal cortex” or “nucleus accumbens” or “globus pallidus” or putamen or “primary cortex”) (Topic) |
| 3 | #2 AND #1 |
| 4 | ((“MR scan” or “Magnetic Resonance Imag*” or “MR imag*” or “Structural MRI” or “functional MRI” or “NMR imag*” or “T1 weighted imag*” or “T1-weighted MRI?” or “tensor-based morphometry” or “T1WI MRI” or fMRI or FMRIB or sMRI or “MRI assessment*” or “MRI stud*” or “MRI analys*” or “MRI-based” or “MRI measure*” or “MRI marker*” or vMRI or “Magnetic Resonance Imag*” or “morphometric MRI” or “MR? Biomarker*”) NEAR/2 (brain* or cerebral or temporal or cortical or subcortical or hippocamp* or parahippocamp* or frontal or parietal or temporoparietal or frontoparietal or cerebell* or intracranial or “gr?y matter” or “white matter” or ventricular or orbitofrontal or bifrontal or cortices or diencephalon or pallidum or amygdala* or frontotemporal or thalamus or subthalamus or neuroanatomical or “third ventricle*” or midbrain or parenchymal or thalami? or subthalami? or “basal ganglia” or prefrontal or temporopolar or “angular gyrus” or “postcentral gyrus” or precuneus or “cingulate gyrus” or “gyrus rectus” or “anterior cingulate” or “posterior cingulate” or “corpus callosum” or insula or claustrum or caudate or “frontal gyrus” or “precentral gyrus” or “supplementary motor area” or “paracentral lobule” or “postcentral gyrus” or pons or “medulla oblongata” or “angular gyrus” or “supramarginal gyrus” or “posterior cingulum” or “motor cortex” or “fusiform gyrus” or occipital or “frontal cortex” or “nucleus accumbens” or “globus pallidus” or putamen or “primary cortex”)) (Topic) |
| 5 | #1 AND #4 |
| 6 | ((“MR scan” or “Magnetic Resonance Imag*” or “MR imag*” or “Structural MRI” or “functional MRI” or “NMR imag*” or “T1 weighted imag*” or “T1-weighted MRI?” or “tensor-based morphometry” or “T1WI MRI” or fMRI or FMRIB or sMRI or “MRI assessment*” or “MRI stud*” or “MRI analys*” or “MRI-based” or “MRI measure*” or “MRI marker*” or vMRI or “Magnetic Resonance Imag*” or “morphometric MRI” or “MR? Biomarker*”) NEAR/5 (atroph* or degenerati* or neurodegenerat* or alteration* or changes or “volume loss” or "loss of volume" or “microstructural degradation”) NEAR/6 (brain* or cerebral or temporal or cortical or subcortical or hippocamp* or parahippocamp* or frontal or parietal or temporoparietal or frontoparietal or cerebell* or intracranial or “gr?y matter” or “white matter” or ventricular or orbitofrontal or bifrontal or cortices or diencephalon or pallidum or amygdala* or frontotemporal or thalamus or subthalamus or neuroanatomical or “third ventricle*” or midbrain or parenchymal or thalami? or subthalami? or “basal ganglia” or prefrontal or temporopolar or “angular gyrus” or “postcentral gyrus” or precuneus or “cingulate gyrus” or “gyrus rectus” or “anterior cingulate” or “posterior cingulate” or “corpus callosum” or insula or claustrum or caudate or “frontal gyrus” or “precentral gyrus” or “supplementary motor area” or “paracentral lobule” or “postcentral gyrus” or pons or “medulla oblongata” or “angular gyrus” or “supramarginal gyrus” or “posterior cingulum” or “motor cortex” or “fusiform gyrus” or occipital or “frontal cortex” or “nucleus accumbens” or “globus pallidus” or putamen or “primary cortex”)) (Topic) |
| 7 | #1 AND #6 |
| 8 | (“Volumetric brain MRI” or “MRI brain volumetry” or “brain MRI” or “Brain Magnetic Resonance” or “Brain MR”) and (brain* or cerebral or temporal or cortical or subcortical or hippocamp* or parahippocamp* or frontal or parietal or temporoparietal or frontoparietal or cerebell* or intracranial or “gr?y matter” or “white matter” or ventricular or orbitofrontal or bifrontal or cortices or diencephalon or pallidum or amygdala* or frontotemporal or thalamus or subthalamus or neuroanatomical or “third ventricle*” or midbrain or parenchymal or thalami? or subthalami? or “basal ganglia” or prefrontal or temporopolar or “angular gyrus” or “postcentral gyrus” or precuneus or “cingulate gyrus” or “gyrus rectus” or “anterior cingulate” or “posterior cingulate” or “corpus callosum” or insula or claustrum or caudate or “frontal gyrus” or “precentral gyrus” or “supplementary motor area” or “paracentral lobule” or “postcentral gyrus” or pons or “medulla oblongata” or “angular gyrus” or “supramarginal gyrus” or “posterior cingulum” or “motor cortex” or “fusiform gyrus” or occipital or “frontal cortex” or “nucleus accumbens” or “globus pallidus” or putamen or “primary cortex”) (Topic) |
| 9 | #8 AND #1 |
| 10 | ((“gr?y matter” or “white matter”) NEAR/0 (loss or damage* or degradation) NEAR/6 (“MR scan” or “Magnetic Resonance Imag*” or “MR imag*” or “Structural MRI” or “functional MRI” or “NMR imag*” or “T1 weighted imag*” or “T1-weighted MRI?” or “tensor-based morphometry” or “T1WI MRI” or fMRI or FMRIB or sMRI or “MRI assessment*” or “MRI stud*” or “MRI analys*” or “MRI-based” or “MRI measure*” or “MRI marker*” or vMRI or “Magnetic Resonance Imag*” or “morphometric MRI” or “MR? Biomarker*”)) |
| 11 | #10 AND #1 |
| 12 | (MRI? or “Magnetic Resonance Imag*” or “MR imag*” or “MR scan*” or “Structural MRI” or “functional MRI” or “NMR imag*” or “T1 weighted imag*” or “T1-weighted MRI?” or “tensor-based morphometry” or “T1WI MRI” or fMRI or FMRIB or sMRI or “MRI assessment*” or “MRI stud*” or “MRI analys*” or “MRI-based” or “MRI measure*” or “MRI marker*” or vMRI or “Magnetic Resonance Imag*” or “morphometric MRI” or “MR? Biomarker*”) (Topic) and ((hypometabolism or metabolic or metabolism or hypermetabolism) NEAR/6 (brain* or cerebral or temporal or cortical or subcortical or hippocamp* or parahippocamp* or frontal or parietal or temporoparietal or frontoparietal or cerebell* or intracranial or “gr?y matter” or “white matter” or ventricular or orbitofrontal or bifrontal or cortices or diencephalon or pallidum or amygdala* or frontotemporal or thalamus or subthalamus or neuroanatomical or “third ventricle*” or midbrain or parenchymal or thalami? or subthalami? or “basal ganglia” or prefrontal or temporopolar or “angular gyrus” or “postcentral gyrus” or precuneus or “cingulate gyrus” or “gyrus rectus” or “anterior cingulate” or “posterior cingulate” or “corpus callosum” or insula or claustrum or caudate or “frontal gyrus” or “precentral gyrus” or “supplementary motor area” or “paracentral lobule” or “postcentral gyrus” or pons or “medulla oblongata” or “angular gyrus” or “supramarginal gyrus” or “posterior cingulum” or “motor cortex” or “fusiform gyrus” or occipital or “frontal cortex” or “nucleus accumbens” or “globus pallidus” or putamen or “primary cortex”)) (Topic) |
| 13 | #1 AND #12 |
| 14 | ((bvFTD or fvFTD or tvFTD or svPPA or nfvPPA or lpPPA or lvPPA or “PPA-L” or PNFA or SemD or “PPA-G” or nfPPA or “PPA-S” or “PSP-RS” or PSPS or “PSP-P” or PAGF or “PSP-PGF” or “PSP-SL” or “PSP-F” or “PSP-bvFTD” or “PSP-PNFA” or “PSP-C” or vPSP or “FTD-MND” or “ALS-MND” or “ALS-FTD” or ALS?FTD or “CBS-CBD” or “CBS-PSP” or “PSP-CBS” or “PSP-PAGF” or “FTLD-TDP” or “FTLD-tau” or sFTLD or gFTLD or sMND or C9FTLD) NEAR/7 (hypometabolism or Hypermetabolism or metabolic or metabolism)) (Topic) or ((bvFTD or fvFTD or tvFTD or svPPA or nfvPPA or lpPPA or lvPPA or “PPA-L” or PNFA or SemD or “PPA-G” or nfPPA or “PPA-S” or “PSP-RS” or PSPS or “PSP-P” or PAGF or “PSP-PGF” or “PSP-SL” or “PSP-F” or “PSP-bvFTD” or “PSP-PNFA” or “PSP-C” or vPSP or “FTD-MND” or “ALS-MND” or “ALS-FTD” or ALS?FTD or “CBS-CBD” or “CBS-PSP” or “PSP-CBS” or “PSP-PAGF” or “FTLD-TDP” or “FTLD-tau” or sFTLD or gFTLD or sMND or C9FTLD) NEAR/5 (atrophy or degeneration or volume* or “changes in structure” or “lower GM density” or “altered functional connectivity” or “WM alteration*” or “WM loss” or “GM loss” or “GM volume*” or “GM atrophy” or “GM change*” or “structural change*” or “microstructural degradation*” or “microstructural alteration*” or “macrostructural atrophy”)) (Topic) |
| 15 | (MRI? or “Magnetic Resonance Imag*” or “MR imag*” or “MR scan*” or “Structural MRI” or “functional MRI” or “NMR imag*” or “T1 weighted imag*” or “T1-weighted MRI?” or “tensor-based morphometry” or “T1WI MRI” or fMRI or FMRIB or sMRI or “MRI assessment*” or “MRI stud*” or “MRI analys*” or “MRI-based” or “MRI measure*” or “MRI marker*” or vMRI or “Magnetic Resonance Imag*” or “morphometric MRI” or “MR? Biomarker*”) (Topic) |
| 16 | #15 AND #14 |
| 17 | ((FDG NEAR/4 PET) or (“FDG? positron emission tomograph*” or FDGPET)) (Topic) |
| 18 | (Fluorodeoxyglucose or "Flu-deoxyglucose" or fludeoxyglucose or "Flu-deoxy-glucose" or "Fluoro Deoxy glucose" or 18F?FDG or "2-[18F]FDG" or "18F-FDG" or 18fdg or "18?F?Fluorodeoxyglucose" or 18Fluorodeoxyglucose or "18F?fluorodeoxyglucose" or "18?F?fluoro-2-deoxy" or "18F? Fluoro-2-Deoxy-D-Glucose" or "2-deoxy-2-[18F]fluoro-D-glucose" or "2 fluoro 2 deoxy d glucose" or "2 fluoro 2 deoxyglucose" or "2 deoxy 2 fluoro d glucose f 18" or "2 deoxy 2 fluoro dextro glucose f 18" or "2 deoxy 2 fluoro glucose f 18" or "2 deoxy 2 fluoroglucose f 18" or "2 deoxy d glucose f 18" or "2 deoxy dextro glucose f 18" or "deoxyfluoroglucose f 18" or "deoxyglucose f 18" or "Fluor-18-FDG" or "fluoro 2 deoxy d glucose f 18" or "fluoro 2 deoxyglucose f 18" or "fluorodeoxy d glucose f 18" or "2 deoxy 2 fluoro d glucose f18" or "2 deoxy 2 fluoro dextro glucose f18" or "2 deoxy 2 fluoro glucose f18" or "2 deoxy 2 fluoroglucose f18" or "2 deoxy d glucose f18" or "2 deoxy dextro glucose f18" or "deoxyfluoroglucose f18" or "deoxyglucose f18" or "fluoro 2 deoxy d glucose f18" or "fluoro 2 deoxyglucose f18" or "fluorodeoxy d glucose f18") (Topic) and (“positron emission tomograph*” or PET) (Topic) = 43,738  17. ((FDG NEAR/4 PET) or (“FDG? positron emission tomograph*” or FDGPET)) (Topic) |
| 19 | #17 OR #18 |
| 20 | #19 AND #1 |
| 21 | #14 AND #19 |
| 22 | ((bvFTD or fvFTD or tvFTD or svPPA or nfvPPA or lpPPA or lvPPA or “PPA-L” or PNFA or SemD or “PPA-G” or nfPPA or “PPA-S” or “PSP-RS” or PSPS or “PSP-P” or PAGF or “PSP-PGF” or “PSP-SL” or “PSP-F” or “PSP-bvFTD” or “PSP-PNFA” or “PSP-C” or vPSP or “FTD-MND” or “ALS-MND” or “ALS-FTD” or ALS?FTD or “CBS-CBD” or “CBS-PSP” or “PSP-CBS” or “PSP-PAGF” or “FTLD-TDP” or “FTLD-tau” or sFTLD or gFTLD or sMND or C9FTLD) NEAR/9 (Fluorodeoxyglucose or "Flu-deoxyglucose" or fludeoxyglucose or "Flu-deoxy-glucose" or "Fluoro Deoxy glucose" or 18F?FDG or "2-[18F]FDG" or "18F-FDG" or 18fdg or "18?F?Fluorodeoxyglucose" or 18Fluorodeoxyglucose or "18F?fluorodeoxyglucose" or "18?F?fluoro-2-deoxy" or "18F? Fluoro-2-Deoxy-D-Glucose" or "2-deoxy-2-[18F]fluoro-D-glucose" or "2 fluoro 2 deoxy d glucose" or "2 fluoro 2 deoxyglucose" or "2 deoxy 2 fluoro d glucose f 18" or "2 deoxy 2 fluoro dextro glucose f 18" or "2 deoxy 2 fluoro glucose f 18" or "2 deoxy 2 fluoroglucose f 18" or "2 deoxy d glucose f 18" or "2 deoxy dextro glucose f 18" or "deoxyfluoroglucose f 18" or "deoxyglucose f 18" or "Fluor-18-FDG" or "fluoro 2 deoxy d glucose f 18" or "fluoro 2 deoxyglucose f 18" or "fluorodeoxy d glucose f 18" or "2 deoxy 2 fluoro d glucose f18" or "2 deoxy 2 fluoro dextro glucose f18" or "2 deoxy 2 fluoro glucose f18" or "2 deoxy 2 fluoroglucose f18" or "2 deoxy d glucose f18" or "2 deoxy dextro glucose f18" or "deoxyfluoroglucose f18" or "deoxyglucose f18" or "fluoro 2 deoxy d glucose f18" or "fluoro 2 deoxyglucose f18" or "fluorodeoxy d glucose f18")) (Topic) and (“positron emission tomograph*” or PET) (Topic) |
| 23 | ((bvFTD or fvFTD or tvFTD or svPPA or nfvPPA or lpPPA or lvPPA or “PPA-L” or PNFA or SemD or “PPA-G” or nfPPA or “PPA-S” or “PSP-RS” or PSPS or “PSP-P” or PAGF or “PSP-PGF” or “PSP-SL” or “PSP-F” or “PSP-bvFTD” or “PSP-PNFA” or “PSP-C” or vPSP or “FTD-MND” or “ALS-MND” or “ALS-FTD” or ALS?FTD or “CBS-CBD” or “CBS-PSP” or “PSP-CBS” or “PSP-PAGF” or “FTLD-TDP” or “FTLD-tau” or sFTLD or gFTLD or sMND or C9FTLD) NEAR/11 (“FDG? positron emission tomograph*” or FDGPET)) (Topic) or ((bvFTD or fvFTD or tvFTD or svPPA or nfvPPA or lpPPA or lvPPA or “PPA-L” or PNFA or SemD or “PPA-G” or nfPPA or “PPA-S” or “PSP-RS” or PSPS or “PSP-P” or PAGF or “PSP-PGF” or “PSP-SL” or “PSP-F” or “PSP-bvFTD” or “PSP-PNFA” or “PSP-C” or vPSP or “FTD-MND” or “ALS-MND” or “ALS-FTD” or ALS?FTD or “CBS-CBD” or “CBS-PSP” or “PSP-CBS” or “PSP-PAGF” or “FTLD-TDP” or “FTLD-tau” or sFTLD or gFTLD or sMND or C9FTLD) NEAR/11 (FDG NEAR/4 PET)) (Topic) |
| 24 | #23 OR #22 OR #21 OR #20 OR #16 OR #13 OR #11 OR #9 OR #7 OR #5 OR # |
| 25 | Limit to: Languages: English. And Exclude Document Types: Meeting Abstract or Editorial Material or Letter or Book Chapters |
