# Supplementary material for: Volumetric MRI and FDG-PET hypometabolism biomarkers of frontotemporal dementia: protocol for a systematic review and meta-analysis
Source: BMJ Open. 2025 Dec 12;15(12):e101729. doi: 10.1136/bmjopen-2025-101729 (PMC12706253; doi:10.1136/bmjopen-2025-101729)
Supplement: Supplementary file 2 [file bmjopen-2025-101729.reviewer_comments.pdf]

PEER REVIEW HISTORY

BMJ Open publishes all reviews undertaken for accepted manuscripts. Reviewers are asked to complete a checklist review form and are provided with free text boxes to elaborate on their assessment. These free text comments are reproduced below.

ARTICLE DETAILS

Title (Provisional)

Volumetric MRI and FDG-PET hypometabolism biomarkers of Frontotemporal Dementia: Protocol for a systematic review and meta-analysis.

Authors

Solomon, Taylor Julie; Antonic-Baker, Ana; Romero, Lorena; Sinclair, Benjamin; O'Brien, Terence J; Vivash, Lucy

VERSION 1 - REVIEW

**Reviewer**	**1**
**Name**	**Mondragón, Jaime**
**Affiliation**	**University of Groningen, Department of Neurology**
**Date**	**30-Jun-2025**
**COI**	**None**

Overall Comment

This manuscript presents a well-structured and scientifically relevant protocol for a systematic review and meta-analysis that aims to evaluate volumetric MRI and FDG-PET hypometabolism as biomarkers in frontotemporal dementia (FTD). The review is specifically focused on differentiating FTD subtypes, with an emphasis on both clinical syndromes and underlying genetic or pathological confirmation. The authors have clearly articulated the rationale for this work, which addresses an important gap in the neuroimaging and dementia biomarker literature. Overall, the protocol is thoughtfully conceived, methodologically sound, and aligns well with the standards expected of such studies.

The authors provide a compelling rationale for their work, noting the absence of a reliable in vivo biomarker that can differentiate between FTD syndromes and neuropathologies. They correctly highlight the potential utility of structural MRI and FDG-PET in refining differential diagnosis, which could have significant implications for clinical management and future therapeutic targeting. The protocol is registered with PROSPERO (CRD42024545302), demonstrating a commitment to transparency and reproducibility.

Major Remarks

1) While the background is strong, the introduction could better define the clinical relevance of differentiating tau vs. TDP-43 pathology using imaging, particularly as this is a stated objective.

2) Consider explicitly linking each question to potential clinical or therapeutic consequences (e.g., utility in trial stratification).

3) The inclusion criteria regarding diagnostic certainty could be elaborated. Although the authors mention that studies will be included regardless of the diagnostic criteria used, it is not entirely clear how they intend to handle variation in the level of diagnostic certainty (e.g., "definite," "probable," or "possible" FTD). It would be useful to understand whether the authors plan to stratify subgroup analyses based on diagnostic certainty or how they will interpret findings from studies with less rigorous diagnostic definitions.

4) While the protocol outlines a valuable set of subgroup analyses, including by genetic mutation (GRN, MAPT, C9orf72) and by pathology (TDP-43 versus tau), it remains unclear whether the authors expect to have sufficient data within each subgroup to support statistically meaningful comparisons. Providing criteria for minimum data thresholds or explaining how sparse subgroup data will be treated (e.g., descriptive synthesis vs. quantitative pooling) would strengthen the methodological rigor of the protocol.

5) The inclusion of both region-of-interest (ROI) and voxel-based morphometry (VBM) approaches in the imaging analysis is a notable strength but raises a methodological question. The authors should clarify how they plan to reconcile differences between these imaging approaches, particularly in terms of spatial resolution and effect size metrics. Are standardization or transformation procedures planned to allow comparison across studies, or will results be analyzed in modality-specific subgroups?

6) One area that would benefit from additional clarification is the approach to regional brain data standardization across included studies. While the protocol appropriately plans to extract imaging data from studies employing various analytical methods, such as region-of-interest volumetry, voxel-based morphometry, and statistical parametric mapping (SPM), it does not specify whether or how regional findings will be harmonized using a standardized anatomical space or atlas. The absence of a stated convention, such as Montreal Neurological Institute (MNI) space, Talairach coordinates, or common anatomical parcellation schemes (e.g., AAL, Desikan-Killiany), may limit the consistency and interpretability of pooled regional results across studies. To enhance methodological transparency and facilitate cross-study comparability, it is recommended that the authors indicate whether they will standardize regional data to a common neuroanatomical framework and describe how studies with different naming conventions or coordinate systems will be reconciled during data extraction and synthesis. Addressing this point would strengthen the protocol and support the reliability of the planned meta-analyses.

7) The protocol also outlines an appropriate risk of bias assessment strategy, using the Cochrane RoB 2.0 tool for randomized trials and the Newcastle-Ottawa Scale for

observational studies. While these tools are standard, it would be helpful for the authors to briefly explain how the results of the quality assessment will influence data synthesis or interpretation. For instance, will studies deemed to be at high risk of bias be excluded from the meta-analysis or included in sensitivity analyses? Briefly describe how these scores will be used (e.g., exclusion, sensitivity analysis).

8) For the data extraction section, please specify how missing data will be handled if not resolved through author contact.

9) The Discussion Section could be slightly expanded to speculate on how the findings might influence biomarker-based trial design or clinical pathways.

Minor Remarks

1) There is some repetition of the study title early in the manuscript, which may be streamlined for clarity.

2) Explicitly stating the four research questions in the abstract (not just summary) would enhance clarity and usefulness for readers scanning the abstract only.

3) In the Strengths and Limitations section, a brief mention of publication/reporting bias or lack of access to individual-level data could provide additional transparency.

4) The literature search strategy, particularly the detailed Boolean queries provided across MEDLINE, EMBASE, and Web of Science, is exhaustive and well-designed. The authors may wish to indicate whether the search will be updated before review completion to ensure comprehensiveness. Consider providing a simplified version of the strategy in the main text with a reference to full search strings in the supplement.

5) Ethics and patient/public involvement are appropriately addressed, though the authors might briefly note whether any patient advocacy groups or individuals with lived experience contributed to the conceptual development of the protocol.

6) The BMJ Open protocol and the PROSPERO registration are highly consistent in scope, inclusion/exclusion criteria, data synthesis strategy, and overall objectives. The protocol manuscript provides more methodological detail, as expected, and includes a slightly broader set of planned analyses (notably the tau vs TDP-43 comparison). I would recommend updating the PROSPERO entry to reflect all four review questions listed in the manuscript for transparency and consistency. No major methodological discrepancies are noted.

7) Indicate the statistical software used and thresholds for acceptance heterogeneity in the Data Synthesis and Analysis Section.

8) Please include the plans for dissemination to public/patient audiences in the Patient and Public Involvement Section.

Overall Remarks

In summary, this protocol outlines a rigorous and much-needed synthesis of the evidence regarding neuroimaging biomarkers in frontotemporal dementia. The use of both structural and functional modalities, the inclusion of genetically/pathologically confirmed cases, and the intent to conduct clinically relevant subgroup analyses are all significant strengths. The authors' careful attention to bias assessment and literature search methodology reflects strong adherence to systematic review standards.

Provided the above clarifications are addressed, particularly concerning subgroup analysis feasibility, imaging heterogeneity, and diagnostic certainty, I believe this protocol is well-positioned to make a valuable contribution to the field.

VERSION 1 - AUTHOR RESPONSE

Major Remarks

1. ***While the background is strong, the introduction could better define the clinical relevance of differentiating tau vs. TDP-43 pathology using imaging, particularly as this is a stated objective.***

We thank the reviewer for highlighting the importance of emphasising the clinical relevance of differentiating tau and TDP-43 pathology using neuroimaging. We have expanded the introduction to explicitly describe how distinguishing between these underlying proteinopathies has meaningful implications for diagnosis, prognosis, and therapeutic interventions in FTD.

The following section has been added in the introduction (page 5):

> "Thus, these neuroimaging modalities offer a timely, non-invasive, readily accessible means of detecting and predicting FTD-specific brain changes indicative of underlying neuropathology. The ability to predict TDP-43 and tau pathology using neuroimaging is crucial as it is likely these proteinopathies require therapeutics with distinct mechanisms of action, thus allowing for precision medicine approaches such as pathology-based treatment. The capacity to classify patients according to their predominant proteinopathies will additionally facilitate accurate patient stratification and enrolment into clinical trials evaluating novel FTD therapeutics."

2. ***Consider explicitly linking each question to potential clinical or therapeutic consequences (e.g., utility in trial stratification).***

We agree with the reviewer that it is important to highlight the clinical and translational implications of each research question. Accordingly, the research questions have been expanded to explicitly link each question to its potential diagnostic, prognostic, or therapeutic relevance, therefore clarifying the translational impact of this research.

The following section has been amended in the introduction (pages 5-6):

(1) "Can regional MRI volumetry and FDG-PET hypometabolism differentiate FTD patients from healthy controls, thereby supporting early and accurate FTD diagnosis upon clinical presentation?"

(2) "Can regional MRI volumetry and FDG-PET hypometabolism differentiate sporadic cases of FTD from healthy controls, improving diagnostic accuracy in the absence of known genetic influence - which accounts for the large majority of cases - and enhancing our understanding of the disease mechanisms underscoring sporadic FTD?"

(3) "Can regional MRI volumetry and FDG-PET hypometabolism differentiate genetic cases of FTD, specifically delineating the three primary genotypes - MAPT, GRN and C9orf72 - most commonly associated with FTD, thus informing genotype-specific disease trajectories and prognosis?"

(4) "Can regional MRI volumetry and FDG-PET hypometabolism differentiate underlying neuropathology, specifically discriminating between tau- and TDP-43-based FTD, hence facilitating appropriate patient stratification in clinical trials and pathology-targeted therapeutic intervention?"

3. ***The inclusion criteria regarding diagnostic certainty could be elaborated. Although the authors mention that studies will be included regardless of the diagnostic criteria used, it is not entirely clear how they intend to handle variation in the level of diagnostic certainty (e.g., "definite," "probable," or "possible" FTD). It would be useful to understand whether***

the authors plan to stratify subgroup analyses based on diagnostic certainty or how they will interpret findings from studies with less rigorous diagnostic definitions.

Variation in diagnostic criteria used and diagnostic certainty across studies are important considerations raised by the reviewer. To address this, we have clarified that analyses will be stratified based on diagnostic criteria and certainty to determine whether these influence the results.

The following sentences have been added to the Data analysis section (page 7):

> "Studies will be incorporated irrespective of diagnostic criteria used and diagnostic certainty categorisation in order to ensure comprehensive coverage of the literature. However, to account for differing levels of diagnostic certainty across studies, we intend to stratify analyses to separately examine FTD cases with a "definite" and "probable" diagnoses, as well as stratifying according to the diagnostic criteria used. This will enable assessment of whether diagnostic criteria and certainty influence biomarker discovery."

4. ***While the protocol outlines a valuable set of subgroup analyses, including by genetic mutation (GRN, MAPT, C9orf72) and by pathology (TDP-43 versus tau), it remains unclear whether the authors expect to have sufficient data within each subgroup to support statistically meaningful comparisons. Providing criteria for minimum data thresholds or explaining how sparse subgroup data will be treated (e.g., descriptive synthesis vs. quantitative pooling) would strengthen the methodological rigor of the protocol.***

We appreciate the reviewer's suggestion to clarify how subgroups with limited data will be handled. Consequently, we have added a clear criterion for minimum subgroup sizes and outlined that smaller subgroups will be synthesised qualitatively rather than quantitatively.

The following sentence has been added to the Data analysis section (page 11):

> "There will be a minimum threshold of five papers per subgroup for inclusion in quantitative analyses; results from subgroups with fewer papers than this will instead be qualitatively synthesised in order to avoid unstable statistical estimates."

5. ***The inclusion of both region-of-interest (ROI) and voxel-based morphometry (VBM) approaches in the imaging analysis is a notable strength but raises a methodological question. The authors should clarify how they plan to reconcile differences between these imaging approaches, particularly in terms of spatial resolution and effect size metrics. Are standardization or transformation procedures planned to allow comparison across studies, or will results be analyzed in modality-specific subgroups?***

We agree that methodological differences between imaging modalities could affect comparability. We have therefore clarified that ROI and VBM data will be quantified separately within their respective modalities. Qualitative analysis will summarise findings across modalities and analysis methods.

The following sentences have been added to the Data analysis section (page 11) in response to both this and the following comment:

> "Analyses will be conducted within imaging modalities, with ROI and VBM findings analysed independently to accounts for methodological differences. To ensure comparability across included studies, analyses will examine ROIs as reported by each study. Thus, we aim to align findings based on equivalent anatomical regions wherever possible as opposed to attempting transformations across differing brain atlas or registration conventions. SPM/VBM findings will be reported following the standard convention, using either coordinates or anatomical label. Regional summaries will also be harmonised by mapping results to the most commonly used brain atlas in the dataset. Moreover, qualitative analysis will summarise findings across modalities and analysis methods."

6. ***One area that would benefit from additional clarification is the approach to regional brain data standardization across included studies. While the protocol appropriately plans to extract imaging data from studies employing various analytical methods, such as region-of-interest volumetry, voxel-based morphometry, and statistical parametric mapping (SPM), it does not specify whether or how regional findings will be harmonized using a standardized anatomical space or atlas. The absence of a stated convention, such as***

Montreal Neurological Institute (MNI) space, Talairach coordinates, or common anatomical parcellation schemes (e.g., AAL, Desikan-Killiany), may limit the consistency and interpretability of pooled regional results across studies. To enhance methodological transparency and facilitate cross-study comparability, it is recommended that the authors indicate whether they will standardize regional data to a common neuroanatomical framework and describe how studies with different naming conventions or coordinate systems will be reconciled during data extraction and synthesis. Addressing this point would strengthen the protocol and support the reliability of the planned meta-analyses.

The reviewer has highlighted the importance of regional standardisation. In response, we have specified that analyses will compare reported ROIs directly and align findings across equivalent anatomical regions, rather than attempt potentially error-prone coordinate transformations across differing atlases. We have additionally clarified that SPM/VBM will be analysed using the reported convention (coordinates or anatomical label). Summaries of anatomical regions will also be investigated, converted to the most commonly used atlas in the dataset

Additions to the text have been made to the Data Analysis section (page 11) as outlined in response to point 5 above.

7. ***The protocol also outlines an appropriate risk of bias assessment strategy, using the Cochrane RoB 2.0 tool for randomized trials and the Newcastle-Ottawa Scale for observational studies. While these tools are standard, it would be helpful for the authors to briefly explain how the results of the quality assessment will influence data synthesis or interpretation. For instance, will studies deemed to be at high risk of bias be excluded from the meta-analysis or included in sensitivity analyses? Briefly describe how these scores will be used (e.g., exclusion, sensitivity analysis).***

We agree that further clarification was required regarding how risk of bias assessments will inform synthesis and interpretation. We have now clearly stated that studies identified as high risk will be retained but subjected to sensitivity analyses to evaluate their impact on overall findings.

The following sentence has been added to the Data analysis section (page 11):

> "Lastly, the risk of bias scores will be incorporated into sensitivity analyses to assess the robustness of the review's findings. Studies with a higher risk of bias will be excluded or analysed separately to determine whether their inclusion significantly influences the overall results and conclusions."

8. ***For the data extraction section, please specify how missing data will be handled if not resolved through author contact.***

We thank the reviewer for raising the issue of missing data. Given the differences in analysis methods and reporting there will doubtless be substantial instances where data available will not include all the intended data extraction variables. The number of studies and subjects included in each analysis will be clearly stated.

The following section has been added to the Data collection section (page 9):

> "In instances of missing data, analysis will proceed using the available data only, with contributing study and participant counts explicitly specified for each analysis."

9. ***The Discussion Section could be slightly expanded to speculate on how the findings might influence biomarker-based trial design or clinical pathways.***

The reviewer has suggested that outlining the potential clinical impact of this research as it relates to clinical trial design is important. Accordingly, the Discussion section has been expanded and now clearly states how this work may inform future biomarker-based clinical trial design.

The following passages have been added to the discussion (pages 12-13):

> "For instance, conducting clinical trials for novel FTD therapeutics presents substantial challenges. The extensive clinical and pathological overlap associated with FTD leads to poor patient stratification and limits the ability to efficiently assess treatment effects[35]. Currently, many clinical trials investigating the efficacy of disease-modifying novel therapeutics targeting tau or TDP-43 recruit individuals with known genetic mutations to increase pathological

certainty within cohorts. Examples include several monoclonal antibody trials, with AL001 tested in GRN-positive cohorts[36], and PBFT-02 tested in GRN- and C9orf72-positive cohorts[37]. Other ongoing studies include tau-targeting therapies such as UCB0107[38] and sodium selenate[39]. To date, these studies have focused on FTD with TDP-43-associated mutations[38] and progressive supranuclear palsy, a primary tauopathy[39]. respectively. Although this approach increases cohort homogeneity, it excludes a significant portion of sporadic FTD patients, reducing the generalisability of study findings and slowing advancements of FTD treatments."

"Therefore, outcomes of this research have direct implications for clinical trial design. The discovery of in vivo neuroimaging biomarkers that differentiate between FTD proteinopathies will facilitate pathology-based trial enrolment, rather than reliance on genetic status. Consequently, such biomarkers would improve stratification of sporadic FTD cases by likely underlying pathology, enabling evaluation of pathology-targeted therapies in individuals whose pathology cannot be inferred with confidence. Furthermore, these biomarkers may enhance endpoint selection, monitoring of disease progression, and accurate detection of drug effects. FTD clinical trials may therefore move towards a precision medicine paradigm that mirrors developments observed in other neurodegenerative diseases."

Minor Remarks

1. ***There is some repetition of the study title early in the manuscript, which may be streamlined for clarity.***

We understand that repetition of the study title may affect readability of the manuscript. In order to enhance clarity and conciseness, all redundant sentences and instances of repetition have been removed or rephrased.

The following sentence in the abstract has been amended (page 2):

> "We propose a protocol for a systematic review and meta-analysis to identify MRI and FDG-PET biomarkers associated with clinical, genetic, and pathological subtypes of FTD."

The following sentence in the introduction has been amended (page 5):

> "This systematic review and meta-analysis will address the following research questions:"

2. ***Explicitly stating the four research questions in the abstract (not just summary) would enhance clarity and usefulness for readers scanning the abstract only.***

We appreciate this suggestion. For brevity, we have listed truncated versions of each research question in the abstract.

The following section has been added to the abstract (page 2):

> "We aim to address the following research questions: Can regional MRI volumetry and FDG-PET hypometabolism differentiate 1) FTD patients from healthy controls; 2) sporadic cases of FTD from healthy controls; 3) genetic cases of FTD (MAPT, GRN, and C9orf72 mutations) and 4) underlying neuropathology, specifically discriminating between tau and TDP-43-based FTD?".

3. ***In the Strengths and Limitations section, a brief mention of publication/reporting bias or lack of access to individual-level data could provide additional transparency.***

We agree that recognising potential publication and reporting bias is pivotal for study transparency. To address this, we have raised this as a potential limitation in both the Strengths and Limitations section, and the discussion.

The following dot point has been added to the Strengths and Limitations section (page 2-3):

> "Publication and/or reporting bias and lack of individual-level data - Studies included for analysis may selectively report significant findings, and the absence of individual-level data limits the ability to independently verify results and harmonise outcomes across studies"

The following sentence has been added to the discussion (page 13):

> "Finally, publication and reporting bias may be present, and the lack of individual-level data restricts the ability to validate reporting consistency between studies."

4. ***The literature search strategy, particularly the detailed Boolean queries provided across MEDLINE, EMBASE, and Web of Science, is exhaustive and well-designed. The authors may wish to indicate whether the search will be updated before review completion to ensure comprehensiveness. Consider providing a simplified version of the strategy in the main text with a reference to full search strings in the supplement.***

We thank the reviewer for recognising our comprehensive search strategy. For completeness, we have included a statement which confirms we intend to update the literature search prior to review completion. We have additionally embedded a simplified version of our search strategy (Table 1) within the main text, with reference to the full search strategy in the supplementary materials. Table 1 can be found at the end of the manuscript document prior to our list of references (page 15).

The following sentences have been added to the Search strategy section (page 7):

> "Representative search terms for each concept are summarised in Table 1, whilst the full search strategies are provided in an online supplementary file."
>
> "In order to ensure the search is comprehensive and exhaustive, the search will be updated before review completion."

5. ***Ethics and patient/public involvement are appropriately addressed, though the authors might briefly note whether any patient advocacy groups or individuals with lived experience contributed to the conceptual development of the protocol.***

We agree with the reviewers observation that patient advocacy groups and patients with lived experience could have contributed to the design of this study protocol. We confirm that this research was developed independently, and this is now communicated in the Patient and Public Involvement section for clarity.

The following section has been amended in the Patient and Public Involvement section (page 11):

> "Neither individuals with lived experience of FTD, nor patient advocacy groups or other community groups were involved in the design, conduct, reporting, or dissemination plans of this research."

6. ***The BMJ Open protocol and the PROSPERO registration are highly consistent in scope, inclusion/exclusion criteria, data synthesis strategy, and overall objectives. The protocol manuscript provides more methodological detail, as expected, and includes a slightly broader set of planned analyses (notably the tau vs TDP-43 comparison). I would recommend updating the PROSPERO entry to reflect all four review questions listed in the manuscript for transparency and consistency. No major methodological discrepancies are noted.***

We thank the reviewer for acknowledging the high level of consistency between the protocol and the PROSPERO registration. In order to uphold such consistency, the research questions stated in our PROSPERO application have been updated (amended 15th October 2025) and are now concordant with the four research questions stated in this protocol paper.

7. ***Indicate the statistical software used and thresholds for acceptance heterogeneity in the Data Synthesis and Analysis Section.***

We thank the reviewer for this point and have incorporated the suggestions.

The following sentence has been added to the Data analysis section (page 10):

> "All analyses will be conducted using Stata, and an I^2 value greater than 75% will be indicative of substantial heterogeneity."

8. ***Please include the plans for dissemination to public/patient audiences in the Patient and Public Involvement Section.***

We have added a sentence describing our intention to communicate our research findings to public and patient audiences following publication.

The following section has been amended in the Patient and Public Involvement section (page 11):

> "However, following publication, dissemination to these audiences may be undertaken to ensure accessibility of research and broader community engagement, such as presentation of research findings at relevant scientific conferences, and other clinical and academic forums. Study findings may also be presented at public-facing events through our institutes and patient advocacy and support groups such as Dementia Australia and Fight Parkinson's Victoria."

9. ***Along with your revised manuscript, please include a copy of the PRISMA-P checklist indicating the page/line numbers of your manuscript where the relevant information can be found***

A PRISMA-P checklist has been uploaded as part of this revised manuscript submission. Page numbers corresponding to where each requirement in the PRISMA-P can be found in this protocol have been included to assist with verification and cross-referencing.
